# Addressing the Chronic Pain–Early Cognitive Decline Comorbidity Among Older Adults: Protocol for the Active Brains Remote Efficacy Trial

**DOI:** 10.2196/47319

**Published:** 2023-09-28

**Authors:** Ana-Maria Vranceanu, Nathaniel R Choukas, Elizabeth A Rochon, Brooke Duarte, Malvina O Pietrzykowski, Katherine McDermott, Julia E Hooker, Ronald Kulich, Yakeel T Quiroz, Robert A Parker, Eric A Macklin, Christine Ritchie, The Active Brains Project, Ryan A Mace

**Affiliations:** 1 Center for Health Outcomes and Interdisciplinary Research Department of Psychiatry, Massachusetts General Hospital Boston, MA United States; 2 Harvard Medical School Boston, MA United States; 3 Department of Anesthesia, Critical Care and Pain Medicine Massachusetts General Hospital Boston, MA United States; 4 Multicultural Alzheimer’s Prevention Program Department of Psychiatry Massachusetts General Hospital Boston, MA United States; 5 Biostatistics Center Massachusetts General Hospital Boston, MA United States; 6 Mongan Institute Center for Aging and Serious Illness and the Division of Palliative Care and Geriatric Medicine Department of Medicine Massachusetts General Hospital Boston, MA United States; 7 Geriatric Medicine Unit Palliative Care & Geriatric Medicine Massachusetts General Hospital Boston, MA United States

**Keywords:** chronic pain, cognitive decline, physical activity, mind-body therapies, aged, telemedicine, randomized clinical trial, mobile phone

## Abstract

**Background:**

Chronic pain and early cognitive decline, which are costly to treat and highly prevalent among older adults, commonly co-occur, exacerbate one another over time, and can accelerate the development and progression of Alzheimer disease and related dementias. We developed the first mind-body activity program (Active Brains [AB]) tailored to the needs of older adults with chronic pain and early cognitive decline. Results from our previous study strongly supported the feasibility of conducting AB remotely and provided evidence for improvements in outcomes.

**Objective:**

We are conducting a single-blinded, National Institutes of Health stage-2, randomized clinical trial to establish the efficacy of AB versus a time-matched and dose-matched education control (Health Enhancement Program [HEP]) in improving self-reported and objective outcomes of physical, cognitive, and emotional functions in 260 participants. The methodology described in this paper was informed by the *lessons learned* from the first year of the trial.

**Methods:**

Participants are identified and recruited through multidisciplinary clinician–referred individuals (eg, pain psychologists and geriatricians), the Rally Research platform, social media, and community partnerships. Interested participants complete eligibility screening and electronic informed consent. Baseline assessments include self-report, performance-based measures (eg, 6-min walk test) and objective measures (eg, Repeatable Battery for the Assessment of Neuropsychological Status). Participants are mailed a wrist-worn ActiGraph device (ActiGraph LLC) to passively monitor objective function (eg, steps) during the week between the baseline assessment and the beginning of the programs, which they continue to wear throughout the programs. After baseline assessments, participants are randomized to either AB or HEP and complete 8 weekly, remote, group sessions with a Massachusetts General Hospital psychologist. The AB group receives a Fitbit (Fitbit Inc) to help reinforce increased activity. Assessments are repeated after the intervention and at the 6-month follow-up. Coprimary outcomes include multimodal physical function (self-report, performance based, and objective). Secondary outcomes are cognitive function (self-report and objective), emotional function, and pain.

**Results:**

We began recruitment in July 2022 and recruited 37 participants across 4 cohorts. Of them, all (n=37, 100%) have completed the baseline assessment, 26 (70%) have completed the posttest assessment, and 9 (24%) are actively enrolled in the intervention (total dropout: n=2, 5%). In the three cohorts (26/37, 70%) that have completed the AB or HEP, 26 (100%) participants completed all 8 group sessions (including minimal makeups), and watch adherence (1937/2072, 93.48%, average across ActiGraph and Fitbit devices) has been excellent. The fourth cohort is ongoing (9/37, 24%), and we plan to complete enrollment by March 2026.

**Conclusions:**

We aim to establish the efficacy of the AB program over a time-matched and dose-matched control in a live video-based trial and test the mechanisms through theoretically driven mediators and moderators. Findings will inform the development of a future multisite effectiveness-implementation trial.

**Trial Registration:**

ClinicalTrials.gov NCT05373745; https://classic.clinicaltrials.gov/ct2/show/NCT05373745

**International Registered Report Identifier (IRRID):**

DERR1-10.2196/47319

## Introduction

### Background

Chronic pain (CP; ie, pain lasting ≥3 mo) and early cognitive decline (ECD; ie, subjective cognitive decline [1] or mild cognitive impairment [2]) commonly co-occur among older adults. An estimated 60% to 75% of people aged >65 years report at least some persistent pain [3]. Older adults with CP are twice as likely to also report ECD [4,5]. The presence of both CP and ECD has an even greater negative impact on older adults’ physical and emotional functioning than either of these conditions separately [6]. Pain impairs cognitive function, accelerates ECD, and is a modifiable risk factor for developing Alzheimer disease and related dementias [5,7]. In contrast, ECD alters pain perception, reduces treatment compliance, and increases the risk for subsequent disability [8]. Consequently, older adults with CP along with ECD (CP-ECD) can become caught in a *disability spiral* whereby cognitive, physical, emotional, and social functioning are interrelated and progressively worsen over time [9,10]. Addressing this comorbidity in the ECD stage is an unexplored opportunity to stop the disability spiral and potentially slow the transition toward dementia.

The current model of treating the CP-ECD comorbidity is incomplete [11]. Medications have limited efficacy [11-15], increase the risk of adverse events such as falls [16], and have harmful side effects that can impair cognition [17]. Walking interventions provide a safe, nonpharmacological alternative for older adults [18,19], with benefits for both CP and ECD [20-22]. However, older adults with CP-ECD face numerous barriers to walking, including pain misconceptions (eg, *hurt equals harm*), maladaptive thoughts (eg, fear avoidance and pain catastrophizing), and rigid regimens (eg, interferes with life and very difficult to implement) [23-26]. Mind-body interventions can directly address these barriers to sustained walking by teaching evidence-based skills (eg, diaphragmatic breathing and mindfulness exercises) to manage CP-ECD and engage in values-based activities. Older adults have increasingly embraced technologies for health care (known as *digital health*), in part owing to the COVID-19 pandemic [27]. Digital health technologies can enhance the delivery, accessibility (eg, live video), and reinforcement of mind-body interventions (eg, Fitbit [Fitbit Inc] reinforcement of walking goals) to address the CP-ECD comorbidity.

**Figure 1:**
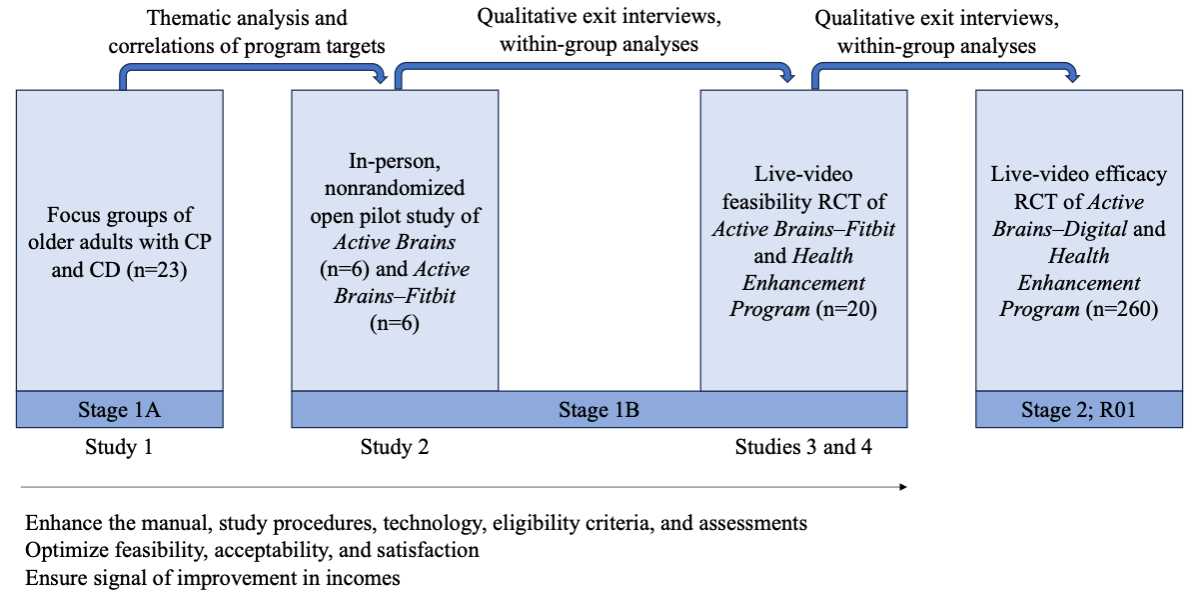
Iterative stages of Active Brains intervention development. The study described in this paper was informed by studies 1 to 4 shown in the figure. CD: cognitive decline; CP: chronic pain; R01: 1R01AG075899-01; RCT: randomized clinical trial.

Our interdisciplinary team has iteratively developed the first web-based, mind-body activity program (Active Brains [AB]) aided by a Fitbit to address the CP-ECD comorbidity among older adults [28]. We followed the National Institutes of Health (NIH) Stage Model [29] and used mixed methods to develop and iteratively optimize the program (Figure 1). We conducted and published the following: (1) qualitative focus groups to understand the needs of older patients with CP-ECD and develop AB [28] (NIH stage 1A); (2) a nonrandomized open pilot study with exit interviews to test the feasibility, satisfaction, and preliminary improvements of AB delivered in-person [30] (NIH stage 1A); (3) adaptation of study procedures for fully remote delivery of AB in response to patient preferences and COVID-19 [31] (NIH stage 1A); and (4) a single-blind feasibility randomized clinical trial (RCT) of AB versus an attention-matched education control (Health Enhancement Program [HEP]), both delivered via live video (NIH stage 1B). Results supported the feasibility, acceptability, credibility, satisfaction, and adherence regarding the web-based AB program and study procedures. Signals of improvement were stronger for AB than for the control in multimodal physical function (step count and self-report) and cognitive function (objective cognitive test and self-reported ECD), emotional function (anxiety), pain intensity, and hypothesized mechanistic targets (mindfulness, coping, catastrophic thoughts about pain, and self-efficacy).

### Objectives

Building on our promising feasibility RCT, we are conducting a web-based RCT of AB versus an attention-matched education control (HEP) in a fully powered study of older adults with CP-ECD (N=260). Randomization is stratified according to ECD type (subjective vs mild cognitive impairment). Our RCT aims to determine the following: (1) efficacy of AB versus HEP on multimodal physical function (coprimary outcomes), cognitive function, emotional function, and pain intensity (secondary outcomes); (2) sustainability of improvements through 6-month follow-up; and (3) mediators (nonadaptive coping, adaptive coping, social factors, and compensatory strategies) and moderators (ECD type, demographics, and clinical variables) of improvement in coprimary and secondary outcomes. To address the lack of diversity in our pilot studies (100% of participants were White) [28,30], our goal is to recruit >38% ethnoracially diverse older adults to align our sample with the US distribution [32]. In this protocol paper, we have described the enhanced methodology, manualized treatments, outcome assessments, current recruitment, and *lessons learned* from the ongoing RCT so far. AB has the potential to be the first evidence-based program for older adults with CP-ECD, with important implications for the prevention of dementia in this high-risk population.

## Methods

### Overview

**Figure 2:**
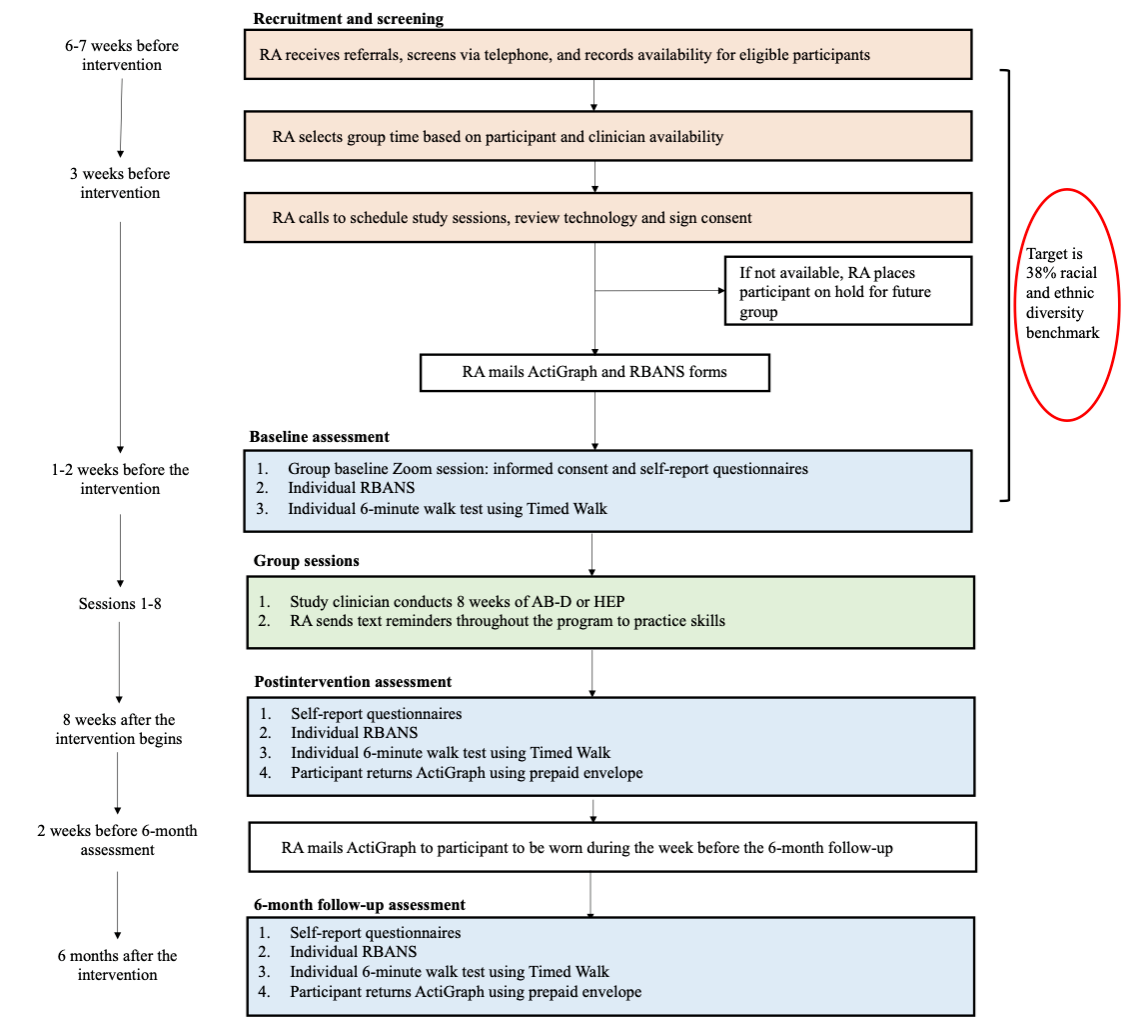
Study design and timeline. AB-D: Active Brains–digital; HEP: Health Enhancement Program; RA: research assistant; RBANS: Repeatable Battery for the Assessment of Neuropsychological Status.

We are conducting this single-blind, NIH stage-2, fully powered, parallel-group efficacy RCT of the AB intervention versus the HEP control at the Massachusetts General Hospital (MGH). Figure 2 depicts the study flow and procedural timeline. We followed live video and technology guidelines for older adults with CP and ECD from our previous protocol [31].

### Ethical Considerations

The Mass General Brigham institutional review board (IRB) approved all the study procedures (2021P002811).

### Participants

Participants are older adults (aged ≥59 y) with self-reported, nonmalignant, musculoskeletal CP (≥3 mo) and ECD (subjective or mild cognitive impairment). Textbox 1 reports full details about the inclusion and exclusion criteria. These eligibility criteria were informed by similar mind-body trials for CP [33,34] and were refined through our previous AB studies [28,30,31,35]. We included older adults with both ECD types (subjective and mild cognitive impairment) because (1) both types increase the risk for dementia among people with CP [7]; (2) our pilot studies included both ECD types, and we did not observe differences in outcomes or skills use [30,35]; (3) it increases generalizability; and (4) it can help to test moderation by ECD type in this fully powered trial.

**Study eligibility criteria and rationale.**

**Inclusion criteria and rationale**

- Aged ≥59 y—population under study
- Have self-identified nonmalignant chronic pain for >3 mo—International Association for the Study of Pain criteria [36]
- Early cognitive decline (subjective or mild cognitive impairment)—both are comorbid with chronic pain and are precursors to dementia
- Telephone Interview for Cognitive Status [37] to confirm the absence of severe cognitive decline that would preclude them from meaningfully engaging with the study
- Functional Activities Questionnaire [38] score <9—to ensure that participants are able to meaningfully engage with the program
- Able to perform a 6-min walk test at an accelerated pace—the Active Brains intervention will involve increasing the number of steps for the coprimary physical function outcome measure
- English fluency and literacy—all measures are validated for use in English
- Ability and willingness to participate and use an ActiGraph watch and smartphone app—the study involves use of multiple technologies, and all participants will receive technological support, but the study requires willingness to learn
- Free of concurrent psychotropic or pain medication for at least 2 wk before initiation of treatment or stable on current psychotropic or pain medication for a minimum of 6 wk and willing to maintain a stable dose—changing pain or psychotropic medication introduces an additional intervention that may act as a third variable

**Exclusion criteria and rationale**

- Diagnosed with dementia or another neurodegenerative disease—these individuals would require a different intervention
- Diagnosed with a medical illness expected to worsen in the next 6 mo (eg, malignancy)—a serious medical illness may act as a third variable
- Active, untreated serious mental illness or substance use disorder—participant safety
- Current, active suicidal ideation—participant safety
- Started a regular mindfulness practice of ≥45 min/wk in the past 6 wk—treatment involves mind-body techniques, and recent initiation of practice would introduce confounds
- Started a course of cognitive behavioral therapy in the last 3 mo—treatment involves cognitive behavioral skills, and recent initiation of treatment would introduce confounds
- Regular use of a digital monitoring device to monitor step count—treatment confound
- Engage in regular exercise for >30 min/d—treatment confound
- Not at risk for falls assessed by the Stopping Elderly Accidents, Deaths, and Injuries Three Key Questions [39,40] or self-reported clearance by a physician for engaging in light physical activity—to ensure the safety of participants

### Recruitment and Screening

We began recruiting participants in July 2022. Older adults with self-reported ECD and CP are recruited from MGH clinics (Pain Clinic, Memory Unit, Multicultural Alzheimer Prevention Program, and Geriatric Medicine Unit) through multidisciplinary clinician–referred individuals (ie, pain psychologists and geriatricians), by distributing our IRB-approved study flyer through hospital-wide emails via the Rally Research web-based platform, and via social media. Our entirely web-based trial enables us to recruit from the local community and nationally (eg, memory cafes and organizations for older adults). Research assistants log all incoming referred individuals in a REDCap (Research Electronic Data Capture; Vanderbilt University) database (for storing protected health information) and a recruitment log (for tracking the flow of referred individuals and contact attempts). Research assistants conduct individual phone screening to provide study details and determine eligibility. We embedded an R code in the recruitment flyer that allows referred individuals to complete the initial eligibility screening on their own via a simple REDCap checklist. Research assistants answer questions about the study and collect information about availability with all eligible referred individuals. Self-reported CP and ECD are confirmed by referring clinicians and a review of the patient’s medical records when available. Referred individuals that do not meet the study criteria are offered a resource sheet for CP and ECD developed for older patients. The principal investigator confirms all the screening before enrollment and consults with the interdisciplinary team of psychologists, neuropsychologists, and geriatricians as needed.

### Inclusion of Ethnoracially Diverse Older Adults

Our team developed strategies to increase the recruitment and retention of ethnoracially diverse older adults. We included recruitment sites within our institution that serve ethnoracially diverse older patients, including the Multicultural Alzheimer Prevention Program and Senior Health. We have consulted with the MGH Community Access, Recruitment, and Engagement Center about a variety of outreach strategies, which include community talks and presentations, tabling, health fairs, individual meetings, and events. We are prioritizing the recruitment of ethnoracially diverse older adults by using recommendations from the literature. These include, but are not limited to, the following: (1) developing culturally sensitive and inclusive study materials, (2) exercising flexibility in scheduling and emphasizing study resources to reduce barriers to participation, (3) asking trusted clinicians (ie, physicians, nurses, social workers, psychologists, and medical assistants) and community members to introduce the study to patients, (4) training the staff on culturally sensitive communication and recruitment strategies that focus on building trust and rapport, (5) prioritizing approaching all ethnoracially diverse older patients who present to recruitment sources as feasible, (6) inviting family into the decision-making process and to support participation (eg, reinforce attendance and troubleshoot technology), (7) addressing the stigma associated with CP and ECD, (8) providing tablets and smartphones with data plans to older adults who do not have them, (9) providing one-on-one training to learn technology, and (10) increasing the diversity of study staff [41-45]. If needed, we will expand recruitment to satellite clinics (MGH Dimmock, Chelsea, Revere, and Waltham) and the Boston Medical Center Pain Clinic.

### Assessments and Enrollment

#### Informed Consent and Mailing of Study Materials

After recruiting at least 8 eligible participants (ie, the minimum threshold to start forming a round of groups), research assistants contact each participant to coordinate the informed consent process and mailing of study procedures. A total of 3 weeks before the first AB or HEP session, research assistants conduct 30-minute Zoom (Zoom Video Communications) meetings with participants to: (1) review and sign the electronic consent form, (2) test Zoom and eliminate barriers to web-based participation, (3) download the Timed Walk app [46] (to self-administer the 6-min walk test), (4) schedule the baseline assessments, (5) assess delivery preferences (timing, email, or SMS text) for receiving daily study reminders, (6) collect an address to mail the study materials, and (7) identify a friend or family member to troubleshoot technology if needed. After consent, a research assistant mails the following to each participant: (1) a study welcome letter, (2) a complementary pen and magnet with the study logo and contact information, (3) Repeatable Battery for the Assessment of Neuropsychological Status (RBANS) Update [47] cognitive assessment forms, and (4) a wrist-worn ActiGraph wGT3X-BT (ActiGraph LLC) [48,49] watch and materials (user-friendly instructions and charger) for blinded step count assessment. We provide tablets (to sync watches, respond to surveys, and attend the AB or HEP sessions) to promote equitable participation of older adults who do not own these devices. These participants receive an additional tutorial with a research assistant to set up their tablet. Participants are instructed to leave the study package unopened until the group, baseline assessment Zoom session. So far, all participants have provided informed consent, and all study procedures have been conducted in accordance with our IRB-approved protocol.

#### Assessments

We selected measures based on the Initiative of Methods, Measurement, and Pain Assessment in Clinical Trials [50] and International Classification of Functioning [51] guidelines for CP clinical trials, our conceptual model [10,52-55], and previous AB studies [28,30]. All outcome measures are assessed at baseline, after the intervention, and at 6-month follow-up. Table 1 reports details about the measures, organized according to construct.

**Table 1:** Study measures and constructs.

Categories and subcategories		Time points
**Demographic factors**		
	Date of birth, gender, weight, height, handedness, race or ethnicity, marital status, education level, employment status, income, and concurrent medical therapies	Baseline
**Clinical factors**		
	Type of ECD^a^ (subjective or objective), pain diagnoses, pain location, length of chronic pain, comorbid medical conditions, current and history of mental health conditions, current pain medication, current memory medication, and current treatments (eg, physiotherapy and occupational therapy)	Baseline
**Coprimary outcomes**		
	Accelerometer (wrist-worn ActiGraph wGT3X-BT)—measures 7-d average step count [48,49]	Baseline, after the intervention, and 6-mo follow-up
	6-min walk test using the Timed Walk app—measures the distance walked in meters at a fast pace for 6 min using smartphone GPS [46]	Baseline, after the intervention, and 6-mo follow-up
	PROMIS^b^ (version 1.2.8b)—measures the level of difficulty with daily function [56,57]	Baseline, after the intervention, and 6-mo follow-up
**Secondary outcomes**		
	Repeatable Battery for the Assessment of Neuropsychological Status—measures 5 cognitive domains (immediate memory, visuospatial and constructional, language, attention, and delayed memory) and global cognition [47]	Baseline, after the intervention, and 6-mo follow-up
	Everyday Cognition Scale—measures self-reported cognitive functioning by comparing current performance on cognitive tasks with that a decade ago [58]	Baseline, after the intervention, and 6-mo follow-up
	PROMIS Depression (version 1.0.8b)—measures negative mood, views of self, engagement in daily living, and social components [59]	Baseline, after the intervention, and 6-mo follow-up
	PROMIS Anxiety (version 1.08a)—measures fear, worry, hyperarousal, and somatic symptoms [59]	Baseline, after the intervention, and 6-mo follow-up
	Numerical Rating Scale—measures pain intensity at rest and during activity during the past week [60]	Baseline, after the intervention, and 6-mo follow-up
	PROMIS Pain Interference (version 1.0.8a)—measures the consequences of pain on relevant aspects of life, including social, cognitive, emotional, physical, and recreational activities [56,61]	Baseline, after the intervention, and 6-mo follow-up
	The Pain, Enjoyment of Life, and General Activity Scale [62]—measures pain intensity and pain interference in the past week	Baseline, after the intervention, and 6-mo follow-up
**Mechanisms**		
	Quota-based pacing—measures the perceived use of quota-based activity pacing using a 4-item scale adapted from the Activity Pacing Questionnaire [63]	Baseline, after the intervention, and 6-mo follow-up
	Memory Compensation Questionnaire—measures the use of cognitive compensatory strategies for actual or perceived memory loss [64,65]	Baseline, after the intervention, and 6-mo follow-up
	Pain Catastrophizing Scale—measures hopelessness, helplessness, and rumination about pain [66]	Baseline, after the intervention, and 6-mo follow-up
	Tampa Kinesiophobia Scale—measures the fear of activity owing to pain or injury [67]	Baseline, after the intervention, and 6-mo follow-up
	Pain Self-Efficacy Questionnaire—measures self-efficacy for performing activities of daily living despite pain [68]	Baseline, after the intervention, and 6-mo follow-up
	Self-Compassion Scale—measures self-compassion in the face of stressors or challenges [69]	Baseline, after the intervention, and 6-mo follow-up
	Measures of Current Status—measures the ability to engage in a series of general coping skills (eg, relaxation, being aware of tension, expressing needs, confidence in coping, and assertiveness) [70]	Baseline, after the intervention, and 6-mo follow-up
	Gratitude Questionnaire—measures the ability to experience gratitude daily [71]	Baseline, after the intervention, and 6-mo follow-up
	Toronto Mindfulness Scale—measures self-reported state-level mindfulness [72]	Baseline, after the intervention, and 6-mo follow-up
**Exploratory**		
	Global Cognitive and Social Engagement—measures the perceived engagement in mentally stimulating social and cognitive activities (involving meaning, learning, challenge, and creativity) and passive activities (eg, watching television)	Baseline, after the intervention, and 6-mo follow-up
	NIH^c^ Toolbox Loneliness Fixed Form Age 18+ (version 2.0)—measures the perception that one is alone, lonely, or socially isolated from others [73]	Baseline, after the intervention, and 6-mo follow-up
	PROMIS Satisfaction with Social Roles and Activities (version 2.0.8a)—measures satisfaction with social roles and responsibilities [74]	Baseline, after the intervention, and 6-mo follow-up
	OQ^d^ Therapeutic Alliance—assesses participant perceptions about therapeutic alliance [75]	After the intervention

^a^ECD: early cognitive decline.

^b^PROMIS: Patient-Reported Outcomes Measurement Information System.

^c^NIH: National Institutes of Health.

^d^OQ: Outcome Questionnaire.

#### Self-Report Measures

Participants complete the self-report measures via the web-based REDCap platform during a 60-minute group assessment via Zoom. In our previous AB studies, we learned that group assessments allow our research assistants to efficiently provide technical support with Zoom (eg, connectivity issues) and REDCap (eg, accessing or responding to survey questions) for multiple participants simultaneously. To encourage focus and privacy during the self-reports, the Zoom host mutes all participants and asks them to use the chat or *raise hand* function for help. After completing the surveys, participants have the option of remaining on the call to receive additional technological support from the research assistants. Research assistants review all self-reports for missing data and errors that were not prevented by the REDCap response validation features.

#### ActiGraph wGT3X-BT

During the group baseline (1-2 wk before the first session), participants open their study package and place the ActiGraph watch on their nondominant wrist to measure step count. Participants are instructed to wear the ActiGraph watch daily (except when bathing) until 1 week after the intervention. Participants sync (using the CentrePoint smartphone app) the watch daily and charge weekly. The research assistants review tips to care for their ActiGraph watch, address common misconceptions (eg, no GPS tracking or on or off switch), and encourage them to contact the study staff for technical support. ActiGraph wGT3X-BT is widely used [49] to measure step count and has demonstrated validity in older adults [76]. Participants receive and wear the ActiGraph watch for 1 more week at the 6-month follow-up and return it in a prepaid envelope.

We estimate an average 7-day step count for each participant, measured by ActiGraph wGT3X-BT at baseline, after the intervention, and at 6-month follow-up using an established protocol [30,34,35]. First, we exclude nonwear (no data) or invalid wear days, which is defined as less than 7 hours of wear time during waking hours [77,78]. Next, we exclude the highest and lowest step-total days to prevent very active or inactive days from skewing average step counts. Finally, we average the remaining valid days (minimum days to calculate average count=3).

#### RBANS Assessment

After the group assessment, participants complete an individual 30-minute RBANS cognitive assessment with trained clinicians via Zoom. The clinician confirms that participants have received the testing materials (RBANS figure copy and coding sheets) and have secured an ideal testing environment. This includes (1) stable connectivity, (2) full-screen Zoom on a tablet or laptop (no smartphone), (3) clear audio, (4) no environmental (phone is silenced and no background activity) distractions, (5) proper lighting, (6) flat surface to write, (7) screen-sharing capability, and (8) privacy for the duration of the test. Following the established telepractice guidelines [79], the clinician shares their screen to show the stimulus book, takes screenshots for scoring, and instructs participants to mail written items (figure drawing and coding) in a prepaid envelope.

#### The 6-Minute Walk Test

After completing RBANS, the clinician instructs the participant to complete the 6-minute walk test outside, on flat terrain using Timed Walk. Timed Walk is a GPS-enabled smartphone app, which enables users to measure walking distance within a fixed time frame. The 6-minute walk test performed using Timed Walk is a valid performance-based measure of physical function and presents a reliable alternative to traditional laboratory assessments [46]. The assessment period is complete once the participants call the research assistant with their 6-minute walk test score.

#### Randomization

A week before the first AB or HEP session, research assistants randomize and enroll participants via phone. Participants are randomly assigned in a 1:1 ratio to AB intervention or HEP control to ensure comparability between groups. The unblinded study biostatistician created the randomization schedule with permuted blocks of random sizes and implemented it in REDCap. Randomization is stratified according to ECD type (subjective vs mild cognitive impairment). To maintain single-arm blinding, study staff and materials refer to AB and HEP as AB1 and AB2, respectfully. Without unblinding, research assistants notify each participant about their group assignment, schedule weekly session times, and provide links to the AB or HEP websites. Research assistants review expectations for attending the group sessions, including the following: (1) timeliness, (2) quiet location, (3) respectfulness of others, (4) no multitasking, (5) wearing the watch, (6) having a pen and the manual ready, and (7) responding to daily smartphone surveys to complete home practice. The research assistant mails a printed copy of the program manual to be received by participants before the first group session. Participants in the AB arm receive a Fitbit to wear, starting after session 1 to ensure that step count assessment remains blind in both groups. Participants are considered to be enrolled after completing session 1 of the AB or HEP arms.

### Treatment Arms

#### Overview

We iteratively developed AB and HEP for the specific needs of older adults with CP-ECD through our previous studies [28,30,31,35]. The control (education only; no skills) is dose-matched and time-matched to the intervention. Both treatment arms are group-based and the intervention is delivered by trained clinicians over 8 weeks (90-min sessions) in addition to patients’ usual care. Participants receive both a paper copy of the structured treatment manual and access to a digital copy through the program website. Both manuals refer to ECD as *memory-related problems* because it was preferred by patients who participated in our qualitative focus groups to develop AB. Participants are encouraged to take notes in the treatment manuals during the sessions and for home practice to promote comprehension and retention. Treatment completers are those who complete a minimum of 6 out of 8 sessions. If participants are unable to attend a session, they are offered a 15-minute makeup with the clinician via Zoom to review missed content and address adherence barriers as needed.

#### AB Intervention

##### Overview

The AB intervention is a multimodal walking program informed by the fear-avoidance model [10]. It integrates relaxation response [52], mindfulness [53], pain-specific cognitive behavioral [54], and operant and physical restoration elements [55]. AB teaches skills to break the *disability spiral* of worsening cognitive, physical, emotional, and social functioning in response to comorbid CP and ECD [9,10]. AB skills include the following: (1) *walking skills* to gradually increase step count through individualized quota-based pacing and goal setting reinforced by a digital monitoring device (Fitbit) linked to valued activities; (2) *mind-body skills* to reduce reactivity to pain or cognitive symptoms through diaphragmatic breathing, body scanning, and mindfulness exercises; (3) *pain-cognition*-*behavior* awareness skills to educate on myths about CP and ECD and understand the disability spiral; (4) *cognitive functioning skills* to develop cognitive compensatory strategies and increase intellectual stimulation; and (5) *social-emotional functioning skills* to cope with stress or setbacks and increase social factors (obtaining social support and decreasing isolation) through self-compassion, gratitude, and acceptance (Table 2). The goal is to provide patients with coping skills to manage pain and develop compensatory strategies to improve their physical, cognitive, and emotional function.

**Table 2:** Session outline for the Active Brains (AB) intervention and Health Enhancement Program (HEP) for older adults with chronic pain (CP) and early cognitive decline.

Session	AB topic	AB skills and session content	HEP topic	HEP session content
1	Taking charge of pain and MRP^a^	Deep breathing	Program overview and CP and MRP	Program goals, understanding CP and MRP, how CP and MRP are connected, and the impact of stress
2	Pace yourself	Mindful breathing and quota-based pacing	The connection among CP, MRP, and physical wellness	The connection between CP and MRP
3	Walk all over pain and MRP	Body scan, mindful walking, and gratitude	Sleep and wellness	Healthy sleeping strategies and cognitive and physical health
4	Mindfulness of pain and MRP	Self-compassion and mindful STOP^b^	Exercise and wellness	Physical exercise, maintaining healthy weight, and tips for becoming active
5	Building brain reserve	Mindful focus	Nutrition 1: the basics	Basic nutrition, portion size and calories, and understanding food labels
6	Staying in the upward spiral	Skill review	Nutrition 2: healthy weight and weight loss	Eating healthy meals and snacks, eating out healthy, and weight loss and BMI
7	Feeling connected with others	Love and kindness mindfulness	Managing your health care for CP and MRP	Communicating with physicians, health diary, medical emergencies, and medication adherence
8	Staying on track and maintaining your progress	Mountain mindfulness	Review of AB	Overview of program content

^a^MRP: memory-related problem.

^b^Stop, Take a breath, Observe with curiosity, Proceed mindfully.

In each session of AB, the clinician and participants review previous session material, discuss home practice, solve barriers, learn educational material, practice program skills and apply them to participants’ daily lives, and create an individualized walking plan (eg, “I will walk to the park once per day while calling family then do housework until I hit my daily goal of 1,200 steps.”) to achieve their step goal. For home practice, participants execute their walking plan, practice skills for at least 5 minutes per day, and log their adherence via daily SMS text messages sent from Twilio [80]. Participants are encouraged to regularly visit the program website to watch videos that explain core concepts (eg, disability spiral and myths about pain) and listen to clinician-guided recordings of mindfulness skills.

##### Fitbit and Quota-Based Pacing

Participants randomized to the AB group receive a Fitbit Inspire [81] to wear from the first session through the 6-month follow-up. Fitbit is a user-friendly and commercially available activity watch that enables participants to monitor (updated step count data on the wrist and Fitbit app) and reinforce (motivating reminders to walk and notifications when step goals are achieved) their daily walking. The AB treatment manual contains written instructions and a tutorial video about wearing, charging, and syncing Fitbit daily. Informed by our internal testing, we instruct participants to wear Fitbit on the nondominant wrist below ActiGraph wGT3X-BT and remove Fitbit during activities with repetitive arm movement (eg, gardening and washing dishes) to prevent step count overinflation. To justify wearing 2 activity watches, we explain that ActiGraph is for the research-grade assessment of step count and Fitbit is to reinforce their quota-based pacing plan.

AB participants are taught to increase their daily step count gradually and safely by following evidence-based guidelines for quota-based pacing [55,82]. The research assistant sets each participant’s initial goal by calculating a truncated average (removing the highest and lowest observation) step count for valid days (≥7 h/d) measured by Fitbit Inspire during the week after session 1. Each subsequent week, the research assistant updates AB participants’ step goal by calculating a weekly truncated average using Fitbit Inspire data synced via the Fitabase web-based platform [83]. AB participants (1) increase their goal by 10% if they achieve their goal, (2) reattempt their goal if they did not achieve their goal for 1 week, or (3) decrease their goal by 10% to 20% if they did not achieve their goal for 2 weeks. Clinicians guide participants in applying AB skills to make the patient’s walking plan noncontingent on CP and ECD symptoms or other barriers to achieving their step goal (eg, weather and time). Research assistants routinely monitor ActiGraph and Fitbit adherence using CentrePoint [84] and Fitabase [83] web-based dashboards, respectively. Participants with 24 hours of nonadherence are contacted for reminders and troubleshooting as needed.

##### Improvements to AB for This Trial

We refined AB based on the results of our pilot feasibility RCT and feedback from participants during exit interviews [35]. First, we streamlined the content in our treatment manual to focus more directly on walking and mindfulness skills. In the updated manual, walking is emphasized as the primary target of AB, and mindfulness is described as a tool to improve coping and support increased activity. Second, we replaced Specific, Measurable, Attainable, Realistic, and Timed (SMART) goals with a simplified walking plan based on feedback from participants stating that the acronym overcomplicated weekly walking goals. Third, we restructured the intervention sessions to incorporate more guided mindfulness practices to support learning before home practice. Similarly, we added structured check-ins at the beginning and summaries at the end of each session to consolidate learning, solve challenges with participants, and encourage skills practice. Fourth, we added information about ActiGraph (for research) and Fitbit (for tracking the daily step goal) to help participants understand the differences between the 2 devices. Fifth, we selected a large font and used a white background with black and dark blue text to maximize contrast, to make the manual more readable for older adults [85]. Sixth, we removed scientific jargon, explained key terms (eg, mindfulness, CP, and ECD) with patient-friendly language, and added figures (eg, to validate that CP-ECD can feel similar to *climbing a mountain* but that gaining AB skills along the way can help). We revised both patient and clinician manuals to ensure that the AB skills are taught to encourage understanding of language in the study outcome measures. Finally, we created animated videos to support the learning of program skills and linked these via a program website throughout the manual.

#### HEP Education Control

Our previous study provides full details about HEP [31]. Briefly, this time-matched and dose-matched attention control accounts for the effects of time spent, feedback, and support from the study clinician and other group members. It consists of lifestyle education consistent with public health recommendations and standards for health promotion (eg, physical activity, sleep, nutrition, healthy weight, and medical appointments). Our team has successfully used HEP as an active control in multiple previous studies [86-88]. We adapted HEP to include population-specific information about CP and ECD symptoms. Consistent with a similar efficacy trial [89], the HEP participants are instructed to journal for 5-10 minutes per day to match the dose of mind-body skill practice in the AB intervention.

### Population-Specific Technology Considerations

#### Overview

Our web-based trial implements several technological solutions to improve the experience of older participants in our study. The treatment arms are delivered via Zoom, which overcomes cost, transportation, and scheduling as important barriers to care in this population. The ActiGraph watch continuously and passively collects step count, which reduces the burden of assessments. Walking goals are reinforced in real time via Fitbit, which addresses difficulty in planning and initiating behavior change with CP and ECD. To bypass ECD symptoms (ie, forgetfulness, distractibility, and difficulty in planning), participants receive SMS text messages to attend sessions, practice program skills, record home practice, and self-monitor pain interference with daily activities. During enrollment, research assistants help AB participants install a shortcut to our program website on their smartphones to easily access clinician-guided recordings of all mindfulness skills. Our pilot study showed that older adults with CP and ECD benefited from and could use these technologies with high feasibility, acceptability, and satisfaction [35].

Our previous study [31] describes our protocol for teaching older adults about technologies based on the National Council on Aging guidelines [90]. During enrollment, research assistants assess participants’ familiarity with technology (eg, experience with Zoom and devices owned), identify individual preferences for remote communication (eg, SMS text, email, or phone), and develop individualized support plans (eg, specific family members who can help). During training sessions, research assistants clarify the value and rationale of each technology, allow time for experimentation, and avoid common pitfalls for communicating with older adults (eg, speaking very loudly or slowly). Our qualitative study has revealed that scheduled, individualized learning sessions; frequent encouragement; and on-call technical support help older participants overcome any initial lack of technological readiness [35].

Informed by *lessons learned* from our pilot RCT, we refined our procedures to further improve participants’ experience with technology. First, we streamlined the written materials in the manual and before-treatment packet on wearing, syncing, and charging the watches. Second, we created a demonstration video about wearing and caring for the watches that participants could easily access on the program website. Third, we increased the involvement of family members and friends to help participants troubleshoot challenges with Zoom, email, and responding to SMS text message surveys. Fourth, participants can receive in-person device setup and training with a research assistant during the baseline period (US $10 is provided for travel). Fifth, participants are encouraged to contact the research assistant with technology-related questions and are offered one-on-one appointments when more extensive troubleshooting or training is required.

#### Treatment Fidelity

We follow the NIH Science of Behavioral Change guidelines to support treatment fidelity [91]. We have developed structured manuals and standard operating procedures that we revised based on the lessons learned after our feasibility pilot study of AB [35] and similar efficacy trials [86]. All study staff meet weekly to discuss participant progress through the study, including session attendance, watch use, home practice adherence, and any deviations from the treatment protocol. All study clinicians are Doctor of Philosophy level clinical psychologists and doctoral-level trainees. The clinicians are directly supervised by the project manager who has extensive training in geriatrics, CP, and mind-body treatments. Following a standardized training protocol, clinicians read selected papers about treating CP and ECD in older adults, listen to audio recordings of the sessions, rehearse the clinician manuals, and colead with a senior clinician before running their first group. Clinicians receive training about adherence to the treatment manual, completion of study forms, assessing participant comprehension, and monitoring the use of skills. During weekly in-person supervision, the clinicians receive feedback about protocol adherence, specific participant or group concerns, and session preparation. All sessions are audio recorded. Clinicians record participant attendance and complete checklists of the content delivered. We will randomly select up to 20% (approximately 40) of these checklists to review. Independent coders will assess each session against a coding scheme to evaluate protocol fidelity, with attention to coder discrepancies. Home practice (eg, step count and skills used) is tracked automatically and reviewed during weekly supervision.

### Data Analytic Plan

#### Power Analysis

Our blinded statistician used SAS (version 9.4; SAS Institute) [92] for the power analysis. Our primary outcome requires clinically important improvements in both functional measures (step count and 6-min walk test) and patient-reported outcome (Patient-Reported Outcomes Measurement Information System Physical Function). Allowing for 20% dropouts, 260 participants recruited (130/group; 104/group completing the study) would provide 92.3% power (Cronbach α=.05; 2 sided) to detect the minimum clinically important difference in step count (1000 steps) [93,94] between the AB and HEP groups in the change from baseline to postintervention assessment. This assumes an SD of 2120, which is the upper 80% confidence limit for the SD observed in our pilot study, as recommended [95]. We also assume 50% correlation between preintervention and postintervention measures. This sample size provides 81.8% power for a difference of 54 meters (the minimum clinically important difference) [96] in the 6-minute walk test (SD 135 based on our pilot data) and >99.9% power for a difference of 5.48 points (minimum clinically important difference) [97] for the Patient-Reported Outcomes Measurement Information System Physical Function score (SD 7.61 based on our pilot data). Assuming a correlation of 0.5 between significant results of step count and 6-minute walk test, the power of the study would be 80.7% (based on simulations) of having a statistically significant result for all 3 coprimary end points.

#### Data Analyses

##### Overview

Our blinded statistician will conduct all data analyses using SAS (version 9.4) [92]. We will estimate treatment-dependent changes in primary (objective, performance-based, and patient-reported physical function) and secondary outcomes (patient-reported and objective cognitive function and emotional function). We will follow the intention-to-treat principle and include all randomized participants in our primary efficacy analyses. Data will be analyzed using a shared-baseline, linear mixed model with fully unstructured covariance among repeated measures. The shared-baseline assumption reflects the true state of the population before randomization and has the benefit of adjusting for chance differences at baseline. Linear mixed models with maximum likelihood estimation are unbiased when data are missing at random, conditional on the observed data. If loss to follow-up is strongly treatment-dependent (relative risk >2), we will use perturbed multiple imputation to assess the robustness of estimates from the primary model if data were missing in not-at-random manner.

##### Aim 1: Efficacy of AB (Baseline to Posttest Period)

We have already established the feasibility benchmarks of the intervention and control [28,30]; therefore, emphasis of this study is on establishing the efficacy of AB*.* Accordingly, the primary analysis will compare the changes in primary and secondary outcomes (continuous measures) from baseline to posttest period between the randomized conditions. For each outcome, we will compare the effect of AB versus control on changes from baseline to the posttest period using linear contrasts. With 3 coprimary outcomes, no adjustment for multiple comparisons is needed; therefore, we will use 2-sided tests at *P*<.05 to declare the superiority of AB, maintaining an overall 5% type-1 error at our primary postintervention time point. The success of the trial will be declared if participation in AB is associated with statistically significant and clinically important improvement from baseline to posttest period over participation in control based on the 3 physical function outcomes.

We will conduct several sensitivity analyses to ensure that a conclusion about efficacy is robust to possible deviations from the assumptions of the primary model. Baseline parameters will be included to account for chance differences owing to randomization and to explain the sources of variation in response that are independent of treatment group. We will also use a Wilcoxon rank sum test comparing the change in scores between the 2 groups to avoid any parametric assumptions about the data. All secondary outcome measures will be analyzed using the same shared-baseline, linear mixed model and the same secondary sensitivity analyses. Step-down, Bonferroni-adjusted *P* values will be used to control for multiple comparisons regarding the secondary outcomes.

##### Aim 2: Sustainability of AB (Posttest Period to Follow-Up)

The same shared-baseline analyses will be used to determine durability (eg, changes between *after the intervention* and *6-month follow-up*) on the coprimary and secondary outcomes. Specifically, the persistence of a benefit from AB at 6-month follow-up will be estimated from linear contrasts of the postintervention period to 6 months least-square means and analyzed as a noninferiority test of durability. Noninferiority of AB in maintaining benefits relative to control will be declared if the lower 1-sided 95% confidence bound for a given coprimary outcome is less negative than each outcome’s minimum clinically important difference in favor of control.

##### Aim 3: Mediation and Moderation

We will explore the extent to which treatment-dependent effects are associated with a priori–defined mediators and moderators. Mediation analyses of postintervention change scores will be conducted following a causal model [98] that extends traditional models for mediation [99,100]. The magnitude of the mediated effect will be estimated as the natural indirect effect, which includes effects mediated by an interaction between the treatment and the mediator [101,102]. This extends the traditional Baron and Kenny method and is equivalent to the analysis of Kraemer et al [103] for the linear models. Tests of significance for natural indirect effect estimates will use 2-tailed *t* tests based on SEs estimated using the delta method. Mediation tests will be conducted separately for changes in outcomes of physical, emotional, and cognitive function and pain intensity. The possible effect of moderators of a beneficial effect of AB will be investigated by adding moderator (eg, gender and subjective vs objective cognitive decline), moderator × treatment, and moderator × treatment × visit interaction terms to the repeated-measures analysis of variance described for aim 1.

## Results

The study was funded by the National Institute on Aging in December 2021. The study was approved by the IRB on December 8, 2021. We began recruitment in July 2022. As of March 2023, a total of 201 individuals have been referred and all have been contacted. Of these 201 individuals, we screened 141 (70.1%) and enrolled 37 (18.4%) participants (n=18, 49% in AB and n=19, 51% in HEP) across 4 group cohorts (largest group: n=10, 27%). All (37/37, 100%) participants completed the baseline assessment. Of the 37 participants, 1 (3%) dropped after the first AB session owing to an unrelated psychiatric hospitalization and 1 (3%) dropped after the second HEP session owing to extreme orofacial pain. In the three cohorts (26/37, 70%) that have completed the AB or HEP, 26 (100%) participants completed all 8 of the group sessions (including minimal makeups) and the posttest assessment. Adherence has been high for the Fitbit and ActiGraph watches, with 93.48% (1937/2072) valid days of wear compliance for both devices across the 2 group cohorts. The fourth group cohort is ongoing (9/37, 24%). We plan to complete enrollment by March 2026 and data analyses by February 2027.

## Discussion

### Significance

Our single-blinded, NIH stage-2 RCT (N=260) is designed to test the efficacy, sustainability, and mechanisms of the AB program in improving self-reported and objective outcomes of physical, cognitive, and emotional function. We anticipate that the results will inform nonpharmacological interventions targeting CP and ECD in older adults. Although most nonpharmacological CP interventions address a discrete pain condition (eg, back pain), we developed our mind-body activity program to address issues that are common across all nonmalignant CP conditions. This is important because most older adults have ≥1 type of pain condition [104]. We are targeting people with both subjective and objective ECD because both are precursors of dementia [1], have similar symptom presentations, and require similar tailoring of skills [105,106]. Our web-based mind-body activity program is grounded in strong theoretical and empirical frameworks [10,52-54], combines mind-body skills with quota-based pacing and engagement in activities of daily living, is delivered in a group format to foster social support, and uses a pacing plan that is tailored to each individual. AB has been iteratively refined using the NIH Stage Model [29] guidelines; follows the Initiative of Methods, Measurement, and Pain Assessment [50] and International Classification of Functioning [51] criteria for CP clinical trials; and has demonstrated excellent feasibility markers and proof of concept [28,30]. The groups conducted so far, although preliminary, show high initial participant retention and adherence to both treatment arms and the study technologies.

This study addresses three key limitations of our pilot studies: (1) recruitment of ethnoracially diverse older adults (38% is the benchmark [32]), (2) assessment of longitudinal follow-up (6 mo), and (3) enhanced methodology for blinded assessment (ActiGraph) and reinforcement (Fitbit) of our step count coprimary outcome. Testing AB versus the education control will yield critical information about the efficacy and durability of improving multimodal physical function in older adults with CP-ECD. We plan to assess the mechanisms for improving the coprimary physical function and secondary outcomes (cognitive function, emotional function, and pain intensity). We will test mediation through theoretically informed constructs (nonadaptive coping, adaptive coping, social factors, and compensatory strategies). We will also test moderation by type of ECD (subjective and objective) and relevant demographic and clinical variables. The results will inform future trials of AB and targets in psychosocial treatments for CP and ECD to optimize physical, emotional, and cognitive outcomes.

### Limitations

Despite our emphasis on refining our RCT methodology, we anticipate several limitations. First, it is challenging to include ethnoracially diverse older adults in clinical trials at our academic medical center. We developed specific strategies to recruit and retain a diverse sample. However, this is our first attempt at implementing these strategies and building trust with new communities of potential participants. Despite plans for future translation studies, the programs are only available in English, which limits diverse groups from participating. Next, we have designed a more *digitally inclusive* trial by providing free tablets, watches, and smartphones with data plans and offering hands-on training to participants who do not have access to or are unfamiliar with these technologies. Some older adults may not be able to meaningfully participate in the web-based group program owing to significant visual, speech, or hearing impairment. In addition, wearing 2 watches may be a burden for some participants. We selected ActiGraph and Fitbit because there were no research-grade accelerometry watches that could remotely toggle between blinded assessment and real-time reinforcement of participants’ step count. Our internal testing and the literature [107-109] show that Fitbit tends to overestimate step count relative to ActiGraph, which has made it challenging to set valid and meaningful quota-based step goals during the intervention. Finally, we risk unblinding participants who find our study on ClinicalTrials.gov [110] database or web-based publications.

### Conclusions

We aim to establish the efficacy of the AB program over a time-matched and dose-matched control in a web-based trial. Our findings from this trial will be used to inform the development of a future multisite, hybrid, effectiveness-implementation trial. We hope to implement AB in routine care for older adults across medical settings and train different disciplines (eg, nurses, social workers, and peer coaches) to deliver it. We plan to culturally adapt the program, through collaborations with community partners, to increase the generalizability of the program and disseminate it to diverse older adults living in the community. AB has the potential to be an effective, low-cost, and sustainable treatment for older adults with CP-ECD, with important implications for the prevention of dementia in this high-risk population.

## Appendix

Multimedia Appendix 1 Peer-reviewer report from the Biobehavioral Medicine and Health Outcomes (BMHO) Study Section - Risk, Prevention and Health Behavior Integrated Review Group - Center for Scientific Review (National Institutes of Health, USA).

## Abbreviations

AB: Active Brains
CP: chronic pain
CP-ECD: chronic pain along with early cognitive decline
ECD: early cognitive decline
HEP: Health Enhancement Program
IRB: institutional review board
MGH: Massachusetts General Hospital
NIH: National Institutes of Health
RBANS: Repeatable Battery for the Assessment of Neuropsychological Status
RCT: randomized clinical trial
REDCap: Research Electronic Data Capture
SMART: Specific, Measurable, Attainable, Realistic, and Timed

## Data Availability

Once the trial is complete, data will be made available upon request.

## References

[1] Verlinden VJ, van der Geest JN, de Bruijn RF, Hofman A, Koudstaal PJ, Ikram MA (2016). Trajectories of decline in cognition and daily functioning in preclinical dementia. Alzheimers Dement.

[2] (2021). What is mild cognitive impairment?. National Institute on Aging.

[3] Molton IR, Terrill AL (2014). Overview of persistent pain in older adults. Am Psychol.

[4] Cao S, Fisher DW, Yu T, Dong H (2019). The link between chronic pain and Alzheimer's disease. J Neuroinflammation.

[5] Whitlock EL, Diaz-Ramirez LG, Glymour MM, Boscardin WJ, Covinsky KE, Smith AK (2017). Association between persistent pain and memory decline and dementia in a longitudinal cohort of elders. JAMA Intern Med.

[6] Weiner DK, Rudy TE, Morrow L, Slaboda J, Lieber S (2006). The relationship between pain, neuropsychological performance, and physical function in community-dwelling older adults with chronic low back pain. Pain Med.

[7] Innes KE, Sambamoorthi U (2020). The potential contribution of chronic pain and common chronic pain conditions to subsequent cognitive decline, new onset cognitive impairment, and incident dementia: a systematic review and conceptual model for future research. J Alzheimers Dis.

[8] van Kooten J, Binnekade TT, van der Wouden JC, Stek ML, Scherder EJ, Husebø BS, Smalbrugge M, Hertogh CM (2016). A review of pain prevalence in Alzheimer's, vascular, frontotemporal and Lewy body dementias. Dement Geriatr Cogn Disord.

[9] Wideman TH, Asmundson GG, Smeets RJ, Zautra AJ, Simmonds MJ, Sullivan MJ, Haythornthwaite JA, Edwards RR (2013). Rethinking the fear avoidance model: toward a multidimensional framework of pain-related disability. Pain.

[10] Vlaeyen JW, Linton SJ (2012). Fear-avoidance model of chronic musculoskeletal pain: 12 years on. Pain.

[11] Cravello L, Di Santo S, Varrassi G, Benincasa D, Marchettini P, de Tommaso M, Shofany J, Assogna F, Perotta D, Palmer K, Paladini A, di Iulio F, Caltagirone C (2019). Chronic pain in the elderly with cognitive decline: a narrative review. Pain Ther.

[12] Mehta D, Jackson R, Paul G, Shi J, Sabbagh M (2017). Why do trials for Alzheimer's disease drugs keep failing? A discontinued drug perspective for 2010-2015. Expert Opin Investig Drugs.

[13] Raina P, Santaguida P, Ismaila A, Patterson C, Cowan D, Levine M, Booker L, Oremus M (2008). Effectiveness of cholinesterase inhibitors and memantine for treating dementia: evidence review for a clinical practice guideline. Ann Intern Med.

[14] Sink KM, Holden KF, Yaffe K (2005). Pharmacological treatment of neuropsychiatric symptoms of dementia: a review of the evidence. JAMA.

[15] Knight R, Khondoker M, Magill N, Stewart R, Landau S (2018). A systematic review and meta-analysis of the effectiveness of acetylcholinesterase inhibitors and memantine in treating the cognitive symptoms of dementia. Dement Geriatr Cogn Disord.

[16] Yoshikawa A, Ramirez G, Smith ML, Foster M, Nabil AK, Jani SN, Ory MG (2020). Opioid use and the risk of falls, fall injuries and fractures among older adults: a systematic review and meta-analysis. J Gerontol A Biol Sci Med Sci.

[17] Wright RM, Roumani YF, Boudreau R, Newman AB, Ruby CM, Studenski SA, Shorr RI, Bauer DC, Simonsick EM, Hilmer SN, Hanlon JT, Health‚ AgingBody Composition Study (2009). Effect of central nervous system medication use on decline in cognition in community-dwelling older adults: findings from the Health, Aging And Body Composition Study. J Am Geriatr Soc.

[18] Prohaska TR, Eisenstein AR, Satariano WA, Hunter R, Bayles CM, Kurtovich E, Kealey M, Ivey SL (2009). Walking and the preservation of cognitive function in older populations. Gerontologist.

[19] Stubbs B, Binnekade TT, Soundy A, Schofield P, Huijnen IP, Eggermont LH (2013). Are older adults with chronic musculoskeletal pain less active than older adults without pain? A systematic review and meta-analysis. Pain Med.

[20] Gheysen F, Poppe L, DeSmet A, Swinnen S, Cardon G, De Bourdeaudhuij I, Chastin S, Fias W (2018). Physical activity to improve cognition in older adults: can physical activity programs enriched with cognitive challenges enhance the effects? A systematic review and meta-analysis. Int J Behav Nutr Phys Act.

[21] Vadalà G, Russo F, De Salvatore S, Cortina G, Albo E, Papalia R, Denaro V (2020). Physical activity for the treatment of chronic low back pain in elderly patients: a systematic review. J Clin Med.

[22] Northey JM, Cherbuin N, Pumpa KL, Smee DJ, Rattray B (2018). Exercise interventions for cognitive function in adults older than 50: a systematic review with meta-analysis. Br J Sports Med.

[23] O'Connor SR, Tully MA, Ryan B, Bleakley CM, Baxter GD, Bradley JM, McDonough SM (2015). Walking exercise for chronic musculoskeletal pain: systematic review and meta-analysis. Arch Phys Med Rehabil.

[24] Slade SC, Patel S, Underwood M, Keating JL (2014). What are patient beliefs and perceptions about exercise for nonspecific chronic low back pain? A systematic review of qualitative studies. Clin J Pain.

[25] Perruchoud C, Buchser E, Johanek LM, Aminian K, Paraschiv-Ionescu A, Taylor RS (2014). Assessment of physical activity of patients with chronic pain. Neuromodulation.

[26] Ambrose KR, Golightly YM (2015). Physical exercise as non-pharmacological treatment of chronic pain: why and when. Best Pract Res Clin Rheumatol.

[27] Mace RA, Mattos MK, Vranceanu AM (2022). Older adults can use technology: why healthcare professionals must overcome ageism in digital health. Transl Behav Med.

[28] Mace RA, Gates MV, Bullard B, Lester EG, Silverman IH, Quiroz YT, Vranceanu AM (2021). Development of a novel mind-body activity and pain management program for older adults with cognitive decline. Gerontologist.

[29] Onken LS, Carroll KM, Shoham V, Cuthbert BN, Riddle M (2014). Reenvisioning clinical science: unifying the discipline to improve the public health. Clin Psychol Sci.

[30] Mace RA, Gates MV, Popok PJ, Kulich R, Quiroz YT, Vranceanu AM (2021). Feasibility trial of a mind-body activity pain management program for older adults with cognitive decline. Gerontologist.

[31] Mace RA, Doorley JD, Popok PJ, Vranceanu AM (2021). Live video adaptations to a mind-body activity program for chronic pain and cognitive decline: protocol for the virtual active brains study. JMIR Res Protoc.

[32] The demographic statistical atlas of the United States - Statistical Atlas. Statistical Atlas.

[33] Wetherell JL, Afari N, Rutledge T, Sorrell JT, Stoddard JA, Petkus AJ, Solomon BC, Lehman DH, Liu L, Lang AJ, Atkinson JH (2011). A randomized, controlled trial of acceptance and commitment therapy and cognitive-behavioral therapy for chronic pain. Pain.

[34] Greenberg J, Lin A, Zale EL, Kulich RJ, James P, Millstein RA, Shapiro H, Schatman ME, Edwards RR, Vranceanu AM (2019). Development and early feasibility testing of a mind-body physical activity program for patients with heterogeneous chronic pain; the GetActive study. J Pain Res.

[35] Doorley JD, Mace RA, Popok PJ, Grunberg VA, Ragnhildstveit A, Vranceanu AM (2022). Feasibility randomized controlled trial of a mind-body activity program for older adults with chronic pain and cognitive decline: the virtual "Active brains" study. Gerontologist.

[36] Merskey H, Bogduk N (1994). Classification of Chronic Pain Descriptions of Chronic Pain Syndromes and Definitions of Pain Terms Second Edition.

[37] Fong TG, Fearing MA, Jones RN, Shi P, Marcantonio ER, Rudolph JL, Yang FM, Kiely DK, Inouye SK (2009). Telephone Interview for Cognitive Status: creating a crosswalk with the Mini-Mental State Examination. Alzheimers Dement.

[38] Pfeffer RI, Kurosaki TT, Harrah CH Jr, Chance JM, Filos S (1982). Measurement of functional activities in older adults in the community. J Gerontol.

[39] Mielenz TJ, Kannoth S, Jia H, Pullyblank K, Sorensen J, Estabrooks P, Stevens JA, Strogatz D (2020). Evaluating a two-level vs. Three-level fall risk screening algorithm for predicting falls among older adults. Front Public Health.

[40] Clinical resources, STEADI—older adult fall prevention. Centers for Disease Control and Prevention.

[41] Ibrahim S, Sidani S (2014). Strategies to recruit minority persons: a systematic review. J Immigr Minor Health.

[42] Otado J, Kwagyan J, Edwards D, Ukaegbu A, Rockcliffe F, Osafo N (2015). Culturally competent strategies for recruitment and retention of African American populations into clinical trials. Clin Transl Sci.

[43] Han H-R, Xu A, Mendez KJW, Okoye S, Cudjoe J, Bahouth M, Reese M, Bone L, Dennison-Himmelfarb C (2021). Exploring community engaged research experiences and preferences: a multi-level qualitative investigation. Res Involv Engagem.

[44] The SHARE approach essential steps of shared decisionmaking: quick reference guide. Agency for Healthcare Research and Quality.

[45] Wells JS, Pugh S, Boparai K, Rearden J, Yeager KA, Bruner DW (2017). Cultural competency training to increase minority enrollment into radiation therapy clinical trials-an NRG oncology RTOG study. J Cancer Educ.

[46] Salvi D, Poffley E, Orchard E, Tarassenko L (2020). The mobile-based 6-minute walk test: usability study and algorithm development and validation. JMIR Mhealth Uhealth.

[47] Randolph C, Tierney MC, Mohr E, Chase TN (1998). The Repeatable Battery for the Assessment of Neuropsychological Status (RBANS): preliminary clinical validity. J Clin Exp Neuropsychol.

[48] ActiGraph wGT3X-BT. ActiGraph.

[49] Barnett A, van den Hoek D, Barnett D, Cerin E (2016). Measuring moderate-intensity walking in older adults using the ActiGraph accelerometer. BMC Geriatr.

[50] Taylor AM, Phillips K, Patel KV, Turk DC, Dworkin RH, Beaton D, Clauw DJ, Gignac MAM, Markman JD, Williams DA, Bujanover S, Burke LB, Carr DB, Choy EH, Conaghan PG, Cowan P, Farrar JT, Freeman R, Gewandter J, Gilron I, Goli V, Gover TD, Haddox JD, Kerns RD, Kopecky EA, Lee DA, Malamut R, Mease P, Rappaport BA, Simon LS, Singh JA, Smith SM, Strand V, Tugwell P, Vanhove GF, Veasley C, Walco GA, Wasan AD, Witter J (2016). Assessment of physical function and participation in chronic pain clinical trials: IMMPACT/OMERACT recommendations. Pain.

[51] International Classification of Functioning, Disability and Health (ICF). World Health Organization.

[52] Park ER, Traeger L, Vranceanu AM, Scult M, Lerner JA, Benson H, Denninger J, Fricchione GL (2013). The development of a patient-centered program based on the relaxation response: the Relaxation Response Resiliency Program (3RP). Psychosomatics.

[53] Kabat-Zinn J (1994). Wherever You Go, There You Are: Mindfulness Meditation in Everyday Life.

[54] McCracken LM (2005). Contextual Cognitive-Behavioral Therapy for Chronic Pain.

[55] Nielson WR, Jensen MP, Karsdorp PA, Vlaeyen JW (2013). Activity pacing in chronic pain: concepts, evidence, and future directions. Clin J Pain.

[56] Stone AA, Broderick JE, Junghaenel DU, Schneider S, Schwartz JE (2016). PROMIS fatigue, pain intensity, pain interference, pain behavior, physical function, depression, anxiety, and anger scales demonstrate ecological validity. J Clin Epidemiol.

[57] PROMIS - Physical function. Shirley Ryan AbilityLab.

[58] Farias ST, Mungas D, Harvey DJ, Simmons A, Reed BR, Decarli C (2011). The measurement of everyday cognition: development and validation of a short form of the Everyday Cognition scales. Alzheimers Dement.

[59] Pilkonis PA, Choi SW, Reise SP, Stover AM, Riley WT, Cella D, PROMIS Cooperative Group (2011). Item banks for measuring emotional distress from the Patient-Reported Outcomes Measurement Information System (PROMIS®): depression, anxiety, and anger. Assessment.

[60] Farrar JT, Young JP, LaMoreaux L, Werth JL, Poole MR (2001). Clinical importance of changes in chronic pain intensity measured on an 11-point numerical pain rating scale. Pain.

[61] Instrument: PROMIS pain interference - short form 6b V1.0. National Institute on Drug Abuse.

[62] Krebs EE, Lorenz KA, Bair MJ, Damush TM, Wu J, Sutherland JM, Asch SM, Kroenke K (2009). Development and initial validation of the PEG, a three-item scale assessing pain intensity and interference. J Gen Intern Med.

[63] Antcliff D, Campbell M, Woby S, Keeley P (2015). Assessing the psychometric properties of an activity pacing questionnaire for chronic pain and fatigue. Phys Ther.

[64] Van der Elst W, Hoogenhout EM, Dixon RA, De Groot RH, Jolles J (2011). The Dutch Memory Compensation Questionnaire: psychometric properties and regression-based norms. Assessment.

[65] Garrett DD, Grady CL, Hasher L (2010). Everyday memory compensation: the impact of cognitive reserve, subjective memory, and stress. Psychol Aging.

[66] Sullivan M, Bishop SR, Pivik JR (1995). The Pain Catastrophizing Scale: development and validation. Psychol Assess.

[67] Monticone M, Ambrosini E, Rocca B, Foti C, Ferrante S (2017). Responsiveness and minimal clinically important changes for the Tampa Scale of Kinesiophobia after lumbar fusion during cognitive behavioral rehabilitation. Eur J Phys Rehabil Med.

[68] Nicholas MK (2007). The pain self-efficacy questionnaire: taking pain into account. Eur J Pain.

[69] Raes F, Pommier E, Neff KD, Van Gucht D (2011). Construction and factorial validation of a short form of the Self-Compassion Scale. Clin Psychol Psychother.

[70] Carver CS (2006). Measure of current status. College of Arts and Sciences Psychology.

[71] Mccullough ME, Emmons RA, Tsang J-A (2002). The grateful disposition: a conceptual and empirical topography. J Pers Soc Psychol.

[72] Lau MA, Bishop SR, Segal ZV, Buis T, Anderson ND, Carlson L, Shapiro S, Carmody J, Abbey S, Devins G (2006). The Toronto Mindfulness Scale: development and validation. J Clin Psychol.

[73] Salsman JM, Butt Z, Pilkonis PA, Cyranowski JM, Zill N, Hendrie HC, Kupst MJ, Kelly MA, Bode RK, Choi SW, Lai JS, Griffith JW, Stoney CM, Brouwers P, Knox SS, Cella D (2013). Emotion assessment using the NIH Toolbox. Neurology.

[74] Hahn EA, Beaumont JL, Pilkonis PA, Garcia SF, Magasi S, DeWalt DA, Cella D (2016). The PROMIS satisfaction with social participation measures demonstrated responsiveness in diverse clinical populations. J Clin Epidemiol.

[75] OQ®-TA - OQ Measures. OQ Measures.

[76] Ngueleu A-M, Barthod C, Best KL, Routhier F, Otis M, Batcho CS (2022). Criterion validity of ActiGraph monitoring devices for step counting and distance measurement in adults and older adults: a systematic review. J Neuroeng Rehabil.

[77] Choi L, Liu Z, Matthews CE, Buchowski MS (2011). Validation of accelerometer wear and nonwear time classification algorithm. Med Sci Sports Exerc.

[78] Schrack JA, Cooper R, Koster A, Shiroma EJ, Murabito JM, Rejeski WJ, Ferrucci L, Harris TB (2016). Assessing daily physical activity in older adults: unraveling the complexity of monitors, measures, and methods. J Gerontol A Biol Sci Med Sci.

[79] Miller TW, Wood JA (2011). Telepractice. Clinical Technologies: Concepts, Methodologies, Tools and Applications.

[80] Communication APIs for SMS, voice, video and authentication. Twilio.

[81] Fitness tracker with heart rate | Shop Fitbit Inspire 2. Fitbit.

[82] Fordyce WE (1984). Behavioural science and chronic pain. Postgrad Med J.

[83] Fitabase - Research device data and analytics. Fitabase.

[84] CentrePoint® Platform | ActiGraph. ActiGraph.

[85] Hou G, Anicetus U, He J (2022). How to design font size for older adults: a systematic literature review with a mobile device. Front Psychol.

[86] Vranceanu AM, Zale EL, Funes CJ, Macklin EA, McCurley J, Park ER, Jordan JT, Lin A, Plotkin SR (2018). Mind-body treatment for international english-speaking adults with neurofibromatosis via live videoconferencing: protocol for a single-blind randomized controlled trial. JMIR Res Protoc.

[87] Reichman M, Riklin E, Macklin E, Vranceanu AM (2020). Virtual mind-body treatment for adolescents with neurofibromatosis: study protocol for a single-blind randomized controlled trial. Contemp Clin Trials.

[88] Vranceanu AM, Riklin E, Merker VL, Macklin EA, Park ER, Plotkin SR (2016). Mind-body therapy via videoconferencing in patients with neurofibromatosis: an RCT. Neurology.

[89] Garland EL, Hanley AW, Nakamura Y, Barrett JW, Baker AK, Reese SE, Riquino MR, Froeliger B, Donaldson GW (2022). Mindfulness-oriented recovery enhancement vs supportive group therapy for co-occurring opioid misuse and chronic pain in primary care: a randomized clinical trial. JAMA Intern Med.

[90] (2022). Resource guide: remote delivery of evidence-based programs. National Council on Aging.

[91] Czajkowski SM, Powell LH, Adler N, Naar-King S, Reynolds KD, Hunter CM, Laraia B, Olster DH, Perna FM, Peterson JC, Epel E, Boyington JE, Charlson ME (2015). From ideas to efficacy: the ORBIT model for developing behavioral treatments for chronic diseases. Health Psychol.

[92] SAS: analytics, artificial intelligence and data management. Statistical Analysis System.

[93] Marshall SJ, Levy SS, Tudor-Locke CE, Kolkhorst FW, Wooten KM, Ji M, Macera CA, Ainsworth BE (2009). Translating physical activity recommendations into a pedometer-based step goal: 3000 steps in 30 minutes. Am J Prev Med.

[94] Demeyer H, Burtin C, Hornikx M, Camillo CA, Van Remoortel H, Langer D, Janssens W, Troosters T (2016). The minimal important difference in physical activity in patients with COPD. PLoS One.

[95] Browne RH (1995). On the use of a pilot sample for sample size determination. Stat Med.

[96] Redelmeier DA, Bayoumi AM, Goldstein RS, Guyatt GH (1997). Interpreting small differences in functional status: the Six Minute Walk test in chronic lung disease patients. Am J Respir Crit Care Med.

[97] Purvis TE, Andreou E, Neuman BJ, Riley LH 3rd, Skolasky RL (2017). Concurrent validity and responsiveness of PROMIS health domains among patients presenting for anterior cervical spine surgery. Spine (Phila Pa 1976).

[98] Valeri L, Vanderweele TJ (2013). Mediation analysis allowing for exposure-mediator interactions and causal interpretation: theoretical assumptions and implementation with SAS and SPSS macros. Psychol Methods.

[99] Baron RM, Kenny DA (1986). The moderator-mediator variable distinction in social psychological research: conceptual, strategic, and statistical considerations. J Pers Soc Psychol.

[100] MacKinnon DP, Lockwood CM, Hoffman JM, West SG, Sheets V (2002). A comparison of methods to test mediation and other intervening variable effects. Psychol Methods.

[101] Ditlevsen S, Christensen U, Lynch J, Damsgaard MT, Keiding N (2005). The mediation proportion: a structural equation approach for estimating the proportion of exposure effect on outcome explained by an intermediate variable. Epidemiology.

[102] Meeker WQ Jr, Cornwell LW, Aroian LA, Kennedy WJ, Odeh RE (1981). Selected Tables in Mathematical Statistics: The Product of Two Normally Distributed Random Variables. Volume VII.

[103] Kraemer HC, Kiernan M, Essex M, Kupfer DJ (2008). How and why criteria defining moderators and mediators differ between the Baron and Kenny and MacArthur approaches. Health Psychol.

[104] Johannes CB, Le TK, Zhou X, Johnston JA, Dworkin RH (2010). The prevalence of chronic pain in United States adults: results of an internet-based survey. J Pain.

[105] Farhang M, Miranda-Castillo C, Rubio M, Furtado G (2019). Impact of mind-body interventions in older adults with mild cognitive impairment: a systematic review. Int Psychogeriatr.

[106] Bannon S, Greenberg J, Mace RA, Locascio JJ, Vranceanu AM (2021). The role of social isolation in physical and emotional outcomes among patients with chronic pain. Gen Hosp Psychiatry.

[107] Feehan L, Clayton C, Carruthers E, Li L (2014). FRI0579-HPR feasibility of using Fitbit Flex to motivate people with rheumatoid arthritis to BE physically active. Ann Rheum Dis.

[108] Garza JL, Wu ZH, Singh M, Cherniack MG (2022). Comparison of the wrist-worn Fitbit Charge 2 and the waist-worn Actigraph GTX3 for measuring steps taken in occupational settings. Ann Work Expo Health.

[109] Gomersall SR, Ng N, Burton NW, Pavey TG, Gilson ND, Brown WJ (2016). Estimating physical activity and sedentary behavior in a free-living context: a pragmatic comparison of consumer-based activity trackers and ActiGraph accelerometry. J Med Internet Res.

[110] ClinicalTrials.gov.

