# Supplementary material for: Addressing the Chronic Pain–Early Cognitive Decline Comorbidity Among Older Adults: Protocol for the Active Brains Remote Efficacy Trial
Source: JMIR Res Protoc. 2023 Sep 28;12:e47319. doi: 10.2196/47319 (PMC10570897; doi:10.2196/47319)
Supplement: Multimedia Appendix 1 [file resprot_v12i1e47319_app1.pdf]

SUMMARY STATEMENT

(Privileged Communication)

PROGRAM CONTACT:
Devon Oskvig
(301) 496-9350
oskvigd@nih.gov

Release Date: **07/02/2021**
Revised Date:

Application Number: **1 R01 AG075899-01**

Principal Investigator

VRANCEANU, ANA-MARIA

Applicant Organization: MASSACHUSETTS GENERAL HOSPITAL

Review Group: **BMHO**
Biobehavioral Medicine and Health Outcomes Study Section

Meeting Date: **06/07/2021**
Council: **OCT 2021**
Requested Start: **09/01/2021**

RFA/PA: **PAR19-070**
PCC: **3DCSCDO**

Project Title: **Addressing the chronic pain-early cognitive decline comorbidity among older adults; The Active Brains study**
SRG Action: **Impact Score:33 Percentile:20**
Next Steps: **Visit https://grants.nih.gov/grants/next_steps.htm**
Human Subjects: **30-Human subjects involved - Certified, no SRG concerns**
Animal Subjects: **10-No live vertebrate animals involved for competing appl.**
Gender: **1A-Both genders, scientifically acceptable**
Minority: **1A-Minorities and non-minorities, scientifically acceptable**
Age: **3A-No children included, scientifically acceptable**

Project Year	Direct Costs Requested	Estimated Total Cost
1	353,968	594,666
2	357,858	601,201
3	358,214	601,799
4	358,214	601,799
5	358,214	601,799
TOTAL	1,786,468	3,001,266

ADMINISTRATIVE BUDGET NOTE: The budget shown is the requested budget and has not been adjusted to reflect any recommendations made by reviewers. If an award is planned, the costs will be calculated by Institute grants management staff based on the recommendations outlined below in the COMMITTEE BUDGET RECOMMENDATIONS section.

1R01AG075899-01 Vranceanu, Ana-Maria

RESUME AND SUMMARY OF DISCUSSION: This application proposes a randomized controlled trial (RCT) to test efficacy, sustainability and examine mechanisms of change of a mind–body activity intervention with digital monitoring for older adults that addresses comorbid chronic pain and early cognitive decline. During discussion, the panel agreed the significance of a multimodal intervention that addresses multiple modifiable risk factors associated with a downward spiral of continuing pain, adverse neurostructural changes, and deteriorating cognitive function is high, with potential to improve health outcomes in a variety of domains and slow the transition to Alzheimer's Disease and Related Dementias(ADRD). Reviewers noted this strong PI and her experienced investigative team's focus on a multimodal program that integrates technology to address the linkage of pain and cognitive decline to functional physical activity is innovative. Additional strengths include preliminary data supporting feasibility and preliminary efficacy, a strong and theoretically-grounded methodology that includes an appropriate control arm, manualized treatment along with evaluation of fidelity and both patient-reported and performance-based assessments. The panel also identified addressable weaknesses such as an exercise program inconsistent with the literature coupled with the absence of an exercise physiologist on the team, limited cognitive assessment and no plan to obtain a clinical diagnosis of mild cognitive impairment, and the lack of assessment of risk factors for ADRD. Overall, the panel agreed the application's strengths outweighed its weaknesses and will have a moderately high impact on improving health outcomes and slowing the transition to Alzheimer's Disease and Related Dementias.

DESCRIPTION (provided by applicant): Chronic pain and early cognitive decline are increasingly prevalent among older adults, bidirectionally related, and individual risk factors for Alzheimer's Disease and Related Dementias (ADRD). Chronic pain and cognitive decline exacerbate each other, placing individuals on a "disability spiral" or worsened physical, emotional, and cognitive functioning with incremental risk of ADRD. Currently there are no evidence-based treatments that successfully address the chronic pain-early cognitive decline comorbidity among older adults. With funding from NCCIH and NIA we used mixed methods to iteratively develop a multimodal, theory grounded, group-based mind-body activity program tailored to the unique needs of older adults with chronic pain and early cognitive decline. The program helps older adults manage pain, increase cognitive reserves, and increase engagement in activities that are meaningful to them using quota-based pacing reinforced by a digital monitoring device. The program shows excellent feasibility and acceptability for virtual delivery and remote data collection. Program participation is associated with improvement in multimodal physical function (self-report, 6-minute walk test and Actigraph measured stepcount), emotional function (depression and anxiety), cognitive function (self-report and objective), pain intensity and theory informed mediators. Building on our promising study we now propose to conduct a fully powered RCT of our mind-body activity program (Active Brains-Digital) versus an educational control (Health Enhancement Program) both delivered virtually among N=260 older adults with musculoskeletal chronic pain and early cognitive decline. We will establish efficacy of Active Brains-Digital, sustainability of improvement over 6 months, and mechanisms of change through relevant mediators and moderators. The trial will take place at the Massachusetts General Hospital using our established methodology and infrastructure. Active Brains-Digital has the potential to be the first evidence-based program for older adults with chronic pain and early cognitive decline, with important implications for prevention of AD/ADRD in this high risk population. This project directly aligns with NIA strategic Goal C, objectives 1 and 3.

PUBLIC HEALTH RELEVANCE: Up to 75% of older adults have at least some persistent pain, and older adults with chronic pain are two times more likely to also report early cognitive decline. Chronic pain and early cognitive decline exacerbate each other, placing individuals on a "disability spiral" or worsened physical, emotional, and cognitive functioning with incremental risk of ADRD. The purpose of

the present investigation is to test the efficacy of a novel, theory informed multimodal mind body activity program to improve physical, emotional and cognitive functioning in older adults with chronic musculoskeletal pain and early cognitive decline. Through this study, we seek to solve the unmet need of lack of evidence-based treatments to address chronic pain in older adults with early cognitive decline through a feasible, acceptable and credible program, and ideally delay progression toward ADRD.

CRITIQUE 1

Significance: 5
Investigator(s): 3
Innovation: 3
Approach: 4
Environment: 1

Overall Impact: In light of evidence from epidemiologic studies suggesting that chronic pain (CP) may be linked with early cognitive decline (ECD) and the development of Alzheimer's Disease and Related Dementias (ADRD), the investigators propose a mind–body and activity program that is walking-based and teaches individuals how to manage pain and build cognitive reserves as a solution to address the CP-ECD comorbidity among older adults. The environment for the study is excellent, housing the Integrated Brain Health Clinical and Research Program and the Multicultural Alzheimer's Prevention Program. The study team is also very good but could benefit from the addition of an exercise physiologist to refine the exercise program. Innovation is good as this is the first multimodal program to address the CP-ECD comorbidity in older adults. A few methodological issues are noted including 1) primary outcomes that are not pain or cognition related, particularly when the study premise is built entirely on this co-morbidity 2) an exercise program (potential max of 10% increase in steps from baseline) that is not consistent with the literature on exercise and cognition 3) inclusion and exclusion criteria in need of refinement and 4) lack of longer-term follow-up when there is a focus on ADRD prevention. Enthusiasm is also diminished by the lack of assessment of other established risk factors for ADRD or neuroinflammation – a likely commonality in CP and ECD that may portend the transition to ADRD. Overall, the project is likely to have moderate impact in the field of ADRD.

1. Significance:

Strengths

- ADRD and chronic pain are prevalent in the elderly and associated with significant economic and societal burden.
- Chronic pain can have significant effects on neurocognitive function. Because the neural systems involved in memory and cognition are closely linked to those involved in pain processing, these systems may affect one another reciprocally disrupting cognitive processing and contributing to a downward spiral of continuing pain, adverse neurostructural changes, and deteriorating cognitive function. Altering this downward trajectory may importantly slow the transition to ADRD.

Weaknesses

- While existing studies support a link between chronic pain and ADRD risk, conclusions are limited by substantial study heterogeneity, limited investigation of certain pain conditions, and methodological concerns.

- Noradrenergic system dysfunction and neuroinflammation resulting from microglial pro-inflammatory activation in brain areas mediating the affective component of pain and cognition have been found to influence both chronic pain and AD. It is not clear that the multimodal program designed for this study, which currently does not measure either of these parameters (or any of the other well known risk factors for ADRD) would have a significant effect upon the development of ADRD.
- Based on the guidelines of exercise in old age recommended by official bodies, exercise modes include aerobic activity, strength training (resistance exercise), flexibility, balance, and coordination. In prior studies, resistance exercise had the highest probability of being the optimal exercise type for slowing cognitive decline in patients with cognitive dysfunction, especially in patients with dementia. Multi-component exercise, however, tended to be most effective in protecting global cognition and executive function in patients with MCI. Thus, it is not clear that simply increasing the step count over 8 weeks would improve cognition over the long-term and decrease the occurrence of ADRD.

2. Investigator(s):

Strengths

- PI is the Founding Director of the Integrated Brain Health Clinical and Research Program with a track record in clinical trials and publications on technology enhanced mind-body and lifestyle interventions.
- Additional expertise in clinical trials, Alzheimer’s disease, geriatric medicine, neuropsychology, multicultural populations, pain medicine, and biostatistics.

Weaknesses

- The study team would benefit from the addition of an exercise physiologist.
- Postdoctoral fellow and unblinded statistician roles appear to be more consistent with that of “other personnel”.

3. Innovation:

Strengths

- First multimodal program to address chronic pain – early cognitive decline in older adults.
- Integration of technology (actigraphy, video meeting platform, virtual MoCA, Timed Walk app) to conduct a virtual clinical trial that if successful, could significantly increase access to care.
- Linking walking with activities of daily living that are valued (e.g. walking with family) to sustain activity over time.

Weaknesses

- Technology and methods by themselves are not particularly novel.
- An assessment of neuroinflammatory biomarkers (CSF) would add novelty.

4. Approach:

Strengths

- Multimodal mind-body activity program that is grounded in the fear avoidance model and integrates walking skills, mindfulness, pain-specific cognitive behavioral awareness, and positive psychology skills.
- Rigorous evaluation of intervention fidelity.
- Attempt to enhance access to care using a virtual approach in entirety.
- Evolution of the methodology based on previous trial experience.
- Preliminary data supporting efficacy of the Active-Brains-Digital intervention on improving physical and cognitive function and decreasing pain.

Weaknesses

- Since the premise of the study is that the multimodal mind-body activity program would decrease pain and slow the transition to ADRD (improve cognition) or address the CP-ECD comorbidity, it is not clear why reductions in pain and improvements in cognition are not the primary outcomes. The rationale provided for not assessing pain as the primary outcome is weak - if in fact, the AB-D program will only moderate pain flare-ups and not reduce overall intensity, it appears that the transition to ADRD will not be moderated.
- It is not clear that increasing steps by 600-1000/day (as the primary exercise method) will be sufficient to slow the progression to ADRD. Consideration should also be given to excluding subjects with > 5000 steps/day as part of the broader exclusion criterion of regular exercise for > 30 minutes a day. Such an approach would require a run-in period of evaluation but would minimize confounding from participants who are active but not formally exercising.
- Because it is a treatment confound, any prior exposure to mindfulness practice or CBT should be an exclusion criterion.
- Inclusion criteria for chronic pain should also be defined by a minimum pain score (e.g. > 3).
- Longer-term follow-up (12 months) is desirable since the study premise is focused on slowing the progression to ADRD. Since enrollment will be completed by year 4, a longer-term follow-up focused on pain and cognitive function is feasible. The transition to dementia could also be assessed in a subset of these older patients enrolled in the first 2 years and followed for an additional 3 years.
- The method of assessing subjective cognitive decline (e.g. Subjective Cognitive Decline Questionnaire) or MCI at baseline is undefined.
- Genetic markers of ADRD should be assessed as treatment confounds.
- Practice effects from use of the MoCA within 8 weeks are not addressed.
- With co-primary physical function outcomes, multiple comparisons are not addressed.

5. Environment:

Strengths

- Integrated Brain Health Clinical and Research Program founded by PI for the study, advancement, and clinical practice of evidenced based mind body and lifestyle interventions.
- Pain Clinic and Memory Unit at MGH serve as referral sources.
- Multicultural Alzheimer’s Prevention Program is a resource for the recruitment of a racially and ethnically diverse population.

Weaknesses

- None noted.

Study Timeline:

Strengths

- Multiple sites for recruitment including Boston area black churches.
- Recruitment: Year 1 - 30, Year 2 – 80, Year 3 – 80, Year 4 – 70.
- Contingency plan in place for recruitment deficiencies.

Weaknesses

- None noted

Protections for Human Subjects:

Acceptable Risks and/or Adequate Protections

- Risks related to exercise are appropriately managed.

Data and Safety Monitoring Plan (Applicable for Clinical Trials Only):

Acceptable

- DSMC with members independent of study team

Inclusion Plans:

- Sex/Gender: Distribution justified scientifically
- Race/Ethnicity: Distribution justified scientifically
- Inclusion/Exclusion Based on Age: Distribution justified scientifically
- 61% female; 64% White, 27% African American, 5% Asian, 1% American Indian,1% Native Hawaiian, 2% More than one race; 17% Hispanic

Vertebrate Animals:

Not Applicable (No Vertebrate Animals)

Biohazards:

Not Applicable (No Biohazards)

Resource Sharing Plans:

Acceptable

Budget and Period of Support:

Recommend as Requested

CRITIQUE 2

Significance: 3
Investigator(s): 2
Innovation: 4
Approach: 3
Environment: 1

Overall Impact: This is a well-written application from a strong scientific team with an excellent environment. Significance and innovation are potentially high: chronic pain and cognitive decline are common in older adults and may together contribute to a disability spiral. The team has previously developed the intervention to be tested based on theory, and has pilot-tested it with the relevant population. The described intervention has the potential to lead to improved outcomes in a variety of relevant domains. The main weakness in significance and innovation is that the minimal review of previous PA interventions (or lack thereof) in this population or similar populations. Overall, the approach is very strong with numerous elements that represent a scientifically rigorous design. Investigators are focusing on recruiting a more racially/ethnic diverse sample than they had in their pilot study. I had some minor concerns/ questions about the approach, including some need for clarification on inclusion criteria, content of the control intervention, and the moderator hypotheses. Finally, I would like to see justification of the use of psychologists to lead this program and/or inclusion of other professionals to lead the program. This could contribute to dissemination efforts later.

1. Significance:

Strengths

- Chronic pain and cognitive decline are common in older adults; together, they may contribute to a “disability spiral.”
- The intervention to be tested, Active Minds, uses mind-body skills to promote physical activity. The target of this intervention is improved physical functioning, cognitive functioning, and emotional functioning. A real strength of this intervention is the possibility that it may lead to improved outcomes in a variety of domains. Further, a walking program may be most accessible to this population (compared to a program that requires gym attendance or equipment). The intervention has been developed based on theory and pilot-tested.

Weaknesses

- There is minimal review of previous physical activity interventions (or lack thereof) in similar populations (i.e., older adults with chronic pain or with cognitive decline). This impedes assessment of significance and innovation.

2. Investigator(s):

Strengths

- PI Dr. Vranceanu has expertise in remote delivery of mind-body interventions, interventions to promote physical activity, and research with people with chronic pain. She was PI on an R34 study to develop and pilot-test the proposed intervention.
- The study team includes expertise in gerontology, chronic pain, mind-body interventions, neuropsychology, health disparities, and biostatistics. The team has worked together on previous projects.

Weaknesses

- Although PI Dr. Vranceanu has previously conducted some lifestyle-oriented trials, it is unclear how much expertise there is on the team on assessing, processing, and analyzing physical activity data.

3. Innovation:

Strengths

- Inclusion of people with a variety of types of chronic pain and a range of cognitive impairment will increase generalizability. As investigators point out, many older adults have multiple sites of chronic pain.
- The intervention combines relaxation and mindfulness skills, cognitive techniques to address cognitive barriers to physical activity, and step goals and activity monitoring.

Weaknesses

- There is minimal review of previous physical activity interventions (or lack thereof) in similar populations. This impedes assessment of significance and innovation.

4. Approach:

Strengths

- Investigators have developed the proposed program (Active Brains) and successfully pilot-tested it. Preliminary data suggest that this group prefers virtual delivery of the intervention. Preliminary data document the overall feasibility of a remote version of Active Brains.
- Investigators plan to use a health education program as a control intervention. This will control for time and attention.
- Investigators will measure outcomes using both patient-reported outcomes and objective step counts and assessment of physical functioning (walk test).
- Investigators have created a patient and a therapist manual, and a plan to assess therapist fidelity to the manual.
- Group-based treatment includes elements tailored to the individual (e.g. step goal).
- Particularly because participants will not be required to have access to a smartphone to enroll in the study, investigators may find that some participants have more trouble with technology than the previous group of participants. However, investigators do provide a comprehensive plan to address barriers related to technology use.
- The study appears to be adequately powered.

Weaknesses

- I had a few clarifications re: inclusion criteria. Will canes be allowed? Excluding people with PAR-Q =>1 means excluding people who are on a medication for blood pressure – which is potentially a lot of people. Can there be some way of including them, at least with their primary care providers' permission? Finally, the exclusion criteria of serious mental illness and substance abuse are not well-defined.
- When thinking about real-world implementation of this program, is it realistic to plan that psychologists will be the ones delivering the program? If not, it would make sense to train and use other professionals in this trial.

- It would be helpful to have more details on HEP to ensure does not overlap with the active intervention.
- It appears that there will be a number of moderator analyses, but there are no specific hypotheses re: “relevant clinical and demographic variables.”

5. Environment:

Strengths

- There appears to be an adequate-sized pool of potential participants at MGH.
- MGH is an excellent clinical and academic site for this work.

Weaknesses

- None noted.

Study Timeline:

Strengths

- Study timeline is very detailed.

Weaknesses

- None noted.

Protections for Human Subjects:

Acceptable Risks and/or Adequate Protections

- Risks are minimal.
- Investigators might provide more detail on the potential for loss of confidentiality in a group treatment.

Data and Safety Monitoring Plan (Applicable for Clinical Trials Only):

Acceptable

- Includes a DSMB.

Inclusion Plans:

- Sex/Gender: Distribution justified scientifically
- Race/Ethnicity: Distribution justified scientifically
- Inclusion/Exclusion Based on Age: Distribution justified scientifically
- Investigators plan to increase recruitment of racial/ethnic minorities relative to their pilot trial -- to 38% of the sample.
- Investigators plan to include people aged 60 and older, and at least 50% of their sample wil be women.

Vertebrate Animals:

Not Applicable (No Vertebrate Animals)

Biohazards:

Not Applicable (No Biohazards)

Resource Sharing Plans:

Acceptable

Budget and Period of Support:

Recommend as Requested

CRITIQUE 3

Significance: 2
Investigator(s): 1
Innovation: 2
Approach: 3
Environment: 1

Overall Impact: This application describes an RCT comparing a comprehensive remote group based behavioral intervention to an active control condition to improve physical functioning in older adults with chronic pain and early cognitive decline. The overall impact of this application is judged to be high due to exceptionally strong pilot data that demonstrates feasibility and preliminary efficacy of the intervention, the experienced investigative team and exceptional environment, and the focus on functional everyday engagement. Few weaknesses were identified and the application addressed many of them, including targeted focus on enrolling a diverse sample. While some limitations in traditional cognitive assessment are inherent in a fully remote study design, the objective cognitive assessment is limited to the MoCA, which may not be sensitive to more subtle improvements. The proposal does not sufficiently describe how the MoCA will be used to identify “early cognitive decline” – is there a cut score that will be used? There are also concerns about nonstandard remote administration of the MoCA (i.e., mailing stimuli to participants is not standard practice for telehealth administration of the MoCA). There is also no effort to obtain a clinical diagnosis of MCI or follow-up adjudication of progression to dementia in order to link to the broader literature.

1. Significance:

Strengths

- Addressing multiple potentially modifiable risk factors (that contribute substantially to QOL) for cognitive decline and ADRD will have considerable impact, even if it does not alter the cognitive course, in this difficult to manage population
- The multimodal intervention has the potential to impact multiple outcome domains simultaneously
- Targeted strategies for increasing inclusion of racial/ethnic minority participants
- Patient population is not restricted to a specific type of pain and early cognitive decline is defined broadly to allow greater generalizability

Weaknesses

- Not having a clearly established level of mild cognitive impairment (defined either subjectively or objectively) will make it difficult to replicate
- Failure to assess conversion to dementia as an outcome

2. Investigator(s):

Strengths

- The investigative team covers all the essential areas of expertise and has a strong track record of collaboration and experience with intervention trials

Weaknesses

- None noted

3. Innovation:

Strengths

- Focusing on the combination of pain and cognitive decline in an at-risk population with an intervention (walking) that has the potential to positively impact both conditions
- The conceptual model integrates cutting edge theoretical principles from several different fields to guide the intervention
- The intervention includes several components that are high yield based on several separate literatures, such as physical activity that is functional and social, thereby increasing participation in daily life.

Weaknesses

- All intervention components are well studied in other populations

4. Approach:

Strengths

- The pilot trial data is very compelling and provides clear evidence of feasibility
- Including both patient reported and performance based assessments where possible substantially increases the rigor of these outcomes
- The experience of the team with remote delivery of the intervention
- Builds upon experience and lessons learned in the pilot trials (need to target minority participants, increase usability of activity monitors, longer follow-up duration and providing participants with smartphones)

Weaknesses

- The procedure for administering the MoCA remotely is not described in sufficient detail to determine if it is in line with recommended practice. Reference to mailing visual stimuli to participants is not standard administration procedure via zoom.
- There is no description of how “MCI” will be identified objectively – beyond scoring above the threshold on the TICS, what is the minimal level of objective cognitive impairment (assuming based on the MoCA?)? what is the minimum level of cognitive complaints needed for inclusion?

- Despite cognition being a secondary outcome, the assessment is quite minimal. A more comprehensive remote cognitive assessment would provide more compelling data regarding potential for delaying progression to dementia
- Clinical assessment of MCI vs Dementia vs normal at the end of the trial would allow for translation of the intervention outcomes to the broader ADRD literature

5. Environment:

Strengths

- The environment is exceptionally well-suited for this trial

Weaknesses

- None identified

Study Timeline:

Strengths

- Very detailed timeline

Weaknesses

- None noted

Protections for Human Subjects:

Acceptable Risks and/or Adequate Protections

- no concerns

Data and Safety Monitoring Plan (Applicable for Clinical Trials Only):

Acceptable

- no concerns

Inclusion Plans:

- Sex/Gender: Distribution justified scientifically
- Race/Ethnicity: Distribution justified scientifically
- Inclusion/Exclusion Based on Age: Distribution justified scientifically
- no concerns

Vertebrate Animals:

Not Applicable (No Vertebrate Animals)

Biohazards:

Not Applicable (No Biohazards)

Resource Sharing Plans:

Acceptable

Authentication of Key Biological and/or Chemical Resources:
Acceptable

Budget and Period of Support:
Recommend as Requested

THE FOLLOWING SECTIONS WERE PREPARED BY THE SCIENTIFIC REVIEW OFFICER TO SUMMARIZE THE OUTCOME OF DISCUSSIONS OF THE REVIEW COMMITTEE, OR REVIEWERS' WRITTEN CRITIQUES, ON THE FOLLOWING ISSUES:

PROTECTION OF HUMAN SUBJECTS: ACCEPTABLE

INCLUSION OF WOMEN PLAN: ACCEPTABLE

INCLUSION OF MINORITIES PLAN: ACCEPTABLE

INCLUSION ACROSS THE LIFESPAN: ACCEPTABLE

COMMITTEE BUDGET RECOMMENDATIONS: The budget was recommended as requested.

Footnotes for 1 R01 AG075899-01; PI Name: Vranceanu, Ana-Maria

NIH has modified its policy regarding the receipt of resubmissions (amended applications).See Guide Notice NOT-OD-18-197 at https://grants.nih.gov/grants/guide/notice-files/NOT-OD-18-197.html. The impact/priority score is calculated after discussion of an application by averaging the overall scores (1-9) given by all voting reviewers on the committee and multiplying by 10. The criterion scores are submitted prior to the meeting by the individual reviewers assigned to an application, and are not discussed specifically at the review meeting or calculated into the overall impact score. Some applications also receive a percentile ranking. For details on the review process, see http://grants.nih.gov/grants/peer_review_process.htm#scoring.

MEETING ROSTER

Biobehavioral Medicine and Health Outcomes Study Section
Risk, Prevention and Health Behavior Integrated Review Group
CENTER FOR SCIENTIFIC REVIEW
BMHO
06/07/2021 - 06/08/2021

Notice of NIH Policy to All Applicants: Meeting rosters are provided for information purposes only. Applicant investigators and institutional officials must not communicate directly with study section members about an application before or after the review. Failure to observe this policy will create a serious breach of integrity in the peer review process, and may lead to actions outlined in NOT-OD-14-073 at https://grants.nih.gov/grants/guide/notice-files/NOT-OD-14-073.html and NOT-OD-15-106 at https://grants.nih.gov/grants/guide/notice-files/NOT-OD-15-106.html, including removal of the application from immediate review.

CHAIRPERSON(S)

FILLINGIM, ROGER B, PHD
DISTINGUISHED PROFESSOR
DEPARTMENT OF COMMUNITY DENTISTRY
AND BEHAVIORAL SCIENCE
COLLEGE OF DENTISTRY
UNIVERSITY OF FLORIDA
GAINESVILLE, FL 32610

MEMBERS

BENJAMIN- NEELON, SARA E, PHD
PROFESSOR
DEPARTMENT OF HEALTH
BEHAVIOR AND SOCIETY
JOHNS HOPKINS SCHOOL OF PUBLIC HEALTH
BALTIMORE, MD 21205

BRUEHL, STEPHEN, PHD
PROFESSOR
DEPARTMENT OF ANESTHESIOLOGY
VANDERBILT UNIVERSITY MEDICAL CENTER
NASHVILLE, TN 37212

BUCK, HARLEAH G, PHD *
PROFESSOR
COLLEGE OF NURSING
UNIVERSITY OF IOWA
IOWA CITY, IA 52242

BURG, MATTHEW M, PHD
PROFESSOR
DEPARTMENT OF INTERNAL MEDICINE
YALE UNIVERSITY SCHOOL OF MEDICINE
NEW HAVEN, CT 06520

CHAYTOR, NAOMI S, PHD *
ASSOCIATE PROFESSOR
DEPARTMENT OF MEDICAL EDUCATION
AND CLINICAL SCIENCES
ELSON S. FLOYD COLLEGE OF MEDICINE
WASHINGTON STATE UNIVERISITY
SPOKANE, WA 99202

COLLOCA, LUANA, PHD *
ASSOCIATE PROFESSOR
DEPARTMENT OF PAIN TRANSLATIONAL SYMPTOM
SCIENCE
SCHOOL OF NURSING
UNIVERSITY OF MARYLAND
BALTIMORE, MD 21201

D'ALONZO, KAREN T, PHD *
ASSOCIATE PROFESSOR
DIVISION OF NURSING SCIENCE
SCHOOL OF NURSING
RUTGERS BIOMEDICAL AND HEALTH SCIENCES
RUTGERS, THE STATE UNIVERSITY OF NEW JERSEY
NEWARK, NJ 07102

DUNNE, EUGENE M, PHD *
ASSISTANT PROFESSOR
DEPARTMENT OF ORAL HEALTH SCIENCES
TEMPLE UNIVERSITY
PHILADELPHIA, PA 19140

FORTIER, MICHELLE, PHD
ASSOCIATE PROFESSOR
SUE AND BILL GROSS SCHOOL OF NURSING
UNIVERSITY OF CALIFORNIA, IRVINE
ORANGE, CA 92868

GALLO, LINDA C, PHD *
PROFESSOR
DEPARTMENT OF PSYCHOLOGY
SAN DIEGO STATE UNIVERSITY
SAN DIEGO, CA 92123

GOLDMAN, MYLA D, MD *
PROFESSOR AND VICE CHAIR
DEPARTMENT OF NEUROLOGY
SCHOOL OF MEDICINE
VIRGINIA COMMONWEALTH UNIVERSITY
RICHMOND, VA 23298

HARPER, FELICITY, PHD
ASSOCIATE PROFESSOR
DEPARTMENT OF ONCOLOGY
KARMANOS CANCER INSTITUTE
WAYNE STATE UNIVERSITY
DETROIT, MI 48201

HASSETT, AFTON L, PSYD
ASSOCIATE PROFESSOR
DEPARTMENT OF ANESTHESIOLOGY
CHRONIC PAIN AND FATIGUE RESEARCH CENTER
UNIVERSITY OF MICHIGAN MEDICAL SCHOOL
ANN ARBOR, MI 48106

KRONISH, IAN M, MD
ASSOCIATE PROFESSOR
DEPARTMENT OF MEDICINE
COLUMBIA UNIVERSITY MEDICAL CENTER
NEW YORK, NY 10032

MATHEW, JOSEPH P, MD
PROFESSOR
DEPARTMENT OF ANESTHESIOLOGY
DUKE UNIVERSITY MEDICAL CENTER
DURHAM, NC 27710

MEYER, JACOB D, PHD *
ASSISTANT PROFESSOR
DEPARTMENT OF KINESIOLOGY
COLLEGE OF HUMAN SCIENCES
IOWA STATE UNIVERSITY
AMES, IA 50011

NAPADOW, VITALY, PHD *
ASSOCIATE PROFESSOR
MARTINOS CENTER FOR BIOMEDICAL IMAGING
MASSACHUSETTS GENERAL HOSPITAL
HARVARD MEDICAL SCHOOL
CHARLESTOWN, MA 02129

PYATAK, ELIZABETH F, PHD *
ASSOCIATE PROFESSOR
DEPARTMENT OF OCCUPATIONAL SCIENCE
AND OCCUPATIONAL THERAPY
HEALTH SCIENCE CAMPUS
UNIVERSITY OF SOUTHERN CALIFORNIA
LOS ANGELES, CA 90089

RHEE, KYUNG E, MD
PROFESSOR
DEPARTMENT OF PEDIATRICS
UNIVERSITY OF CALIFORNIA, SAN DIEGO
LA JOLLA, CA 92093

RINI, CHRISTINE, PHD
PROFESSOR
DEPARTMENT OF MEDICAL SOCIAL SCIENCES
FEINBERG SCHOOL OF MEDICINE
NORTHWESTERN UNIVERSITY
CHICAGO, IL 60611

ROPER, JENEVIEVE L, PHD *
ASSISTANT PROFESSOR
DEPARTMENT OF HEALTH AND HUMAN SCIENCES
COLLEGE OF SCIENCE AND ENGINEERING
LOYOLA MARYMOUNT UNIVERSITY
LOS ANGELES, CA 90045

SALWEN-DEREMER, JESSICA K, PHD *
ASSISTANT PROFESSOR
PSYCHIATRY AND MEDICINE
GEISEL SCHOOL OF MEDICINE
DARTMOUTH COLLEGE
HANOVER, NH 03755

SCHEMBRE, SUSAN, PHD *
ASSOCIATE PROFESSOR
DEPARTMENT OF FAMILY AND COMMUNITY MEDICINE
COLLEGE OF MEDICINE TUCSON
THE UNIVERSITY OF ARIZONA HEALTH SCIENCES
TUCSON, AZ 85714

SCHENKER, YAEL, MD *
PROFESSOR OF MEDICINE
SECTION OF PALLIATIVE CARE AND MEDICAL ETHICS
DIVISION OF GENERAL INTERNAL MEDICINE
UNIVERSITY OF PITTSBURGH
PITTSBURGH, PA 15261

SCIAMANNA, CHRISTOPHER N, MD, MPH *
PROFESSOR OF MEDICINE AND PUBLIC HEALTH SERVICES
DIVISION OF GENERAL INTERNAL MEDICINE
MILTON S HERSHEY COLLEGE OF MEDICINE
PENNSYLVANIA STATE UNIVERSITY
HERSHEY, PA 17033

SEN, SOUVIK, MD, MPH *
PROFESSOR AND CENTER FOR ECONOMIC EXCELLENCE
STROKE CHAIR
DEPARTMENT OF NEUROLOGY
UNIVERSITY OF SOUTH CAROLINA
COLUMBIA, SC 29203

SMITH, PATRICK J, PHD
ASSOCIATE PROFESSOR
DEPARTMENT OF PSYCHIATRY AND BEHAVIORAL
SCIENCES
MEDICINE AND POPULATION HEALTH SCIENCES
DUKE UNIVERSITY
DURHAM, NC 27710

SMITH, SAKIMA A, MD *
ASSOCIATE PROFESSOR
DIVISION OF CARDIOVASCULAR MEDICINE
DEPARTMENT OF INTERNAL MEDICINE
WEXNER MEDICAL CENTER
THE OHIO STATE UNIVERSITY
COLUMBUS, OH 43210

TEMPLE, JENNIFER L, PHD
PROFESSOR
DEPARTMENTS OF EXERCISE AND NUTRITION SCIENCES
AND COMMUNITY HEALTH AND HEALTH BEHAVIOR
SCHOOL OF PUBLIC HEALTH AND HEALTH PROFESSIONS
UNIVERSITY AT BUFFALO
BUFFALO, NY 14214

UEBELACKER, LISA A, PHD
PROFESSOR
DEPARTMENT OF PSYCHIATRY AND HUMAN BEHAVIOR
BUTLER HOSPITAL
BROWN UNIVERSITY
PROVIDENCE, RI 02906

VAN CLEAVE, JANET H, PHD *
ASSISTANT PROFESSOR
RORY MEYERS COLLEGE OF NURSING
NEW YORK UNIVERSITY
NEW YORK, NY 10010

VOLLMER, TIMOTHY LEE, MD *
PROFESSOR
DEPARTMENT OF NEUROLOGY
ROCKY MOUNTAIN MS CLINIC ANSCHUTZ MEDICAL CENTER
SCHOOL OF MEDICINE
UNIVERSITY OF COLORADO DENVER
AURORA, CO 80045

WEN, KUANG-YI, PHD *
ASSOCIATE PROFESSOR
SIDNEY KIMMEL CANCER CENTER
THOMAS JEFFERSON UNIVERSITY
PHILADELPHIA, PA 19107

MAIL REVIEWER(S)

PALESH, OXANA G, PHD
ASSOCIATE PROFESSOR
DEPARTMENT OF PSYCHIATRY AND BEHAVIORAL
SCIENCES
STANFORD UNIVERSITY SCHOOL OF MEDICINE
STANFORD, CA 94305

SCIENTIFIC REVIEW OFFICER

VOSVICK, MARK A, PHD
SCIENTIFIC REVIEW OFFICER
CENTER FOR SCIENTIFIC REVIEW
NATIONAL INSTITUTES OF HEALTH
BETHESDA, MD 20892

EXTRAMURAL SUPPORT ASSISTANT

WATTS, MELISSA D
EXTRAMURAL SUPPORT ASSISTANT
CENTER FOR SCIENTIFIC REVIEW
NATIONAL INSTITUTE FOR HEALTH
BETHESDA, MD 20892

* Temporary Member. For grant applications, temporary members may participate in the entire meeting or may review only selected applications as needed.

Consultants are required to absent themselves from the room during the review of any application if their presence would constitute or appear to constitute a conflict of interest.
